# Task-sharing for non-communicable disease prevention and control in low- and middle-income countries in the context of health worker shortages: A systematic review

**DOI:** 10.1371/journal.pgph.0004289

**Published:** 2025-04-16

**Authors:** Azeb Gebresilassie Tesema, Sikhumbuzo A. Mabunda, Kanika Chaudhri, Anthony Sunjaya, Samuel Thio, Kenneth Yakubu, Ragavi Jeyakumar, Myron Godinho, Renu John, Mai Eltigany, Martyna Hogendorf, Rohina Joshi

**Affiliations:** 1 School of Population Health, UNSW, Sydney, Australia; 2 The George Institute for Global Health, UNSW, Sydney, Australia; 3 Department of Public Health, Walter Sisulu University, Mthatha, South Africa; 4 Westmead Applied Research Centre, University of Sydney, Australia; 5 The George Institute for Global Health, UNSW, Delhi, India; 6 World Health Organization, Geneva, Switzerland.; Indian Council of Medical Research, INDIA

## Abstract

Health workers are pivotal for non-communicable disease (NCD) service delivery, yet often are unavailable in low- and middle-income countries (LMICs). There is limited evidence on what NCD-related tasks non-physician health workers (NPHWs) can perform and their effectiveness. This study aims to understand how task-sharing is used to improve NCD prevention and control in LMICs. We also explored barriers, facilitators, and unexpected consequences of task-sharing. Databases searched in two phases and included MEDLINE, EMBASE, CENTRAL, CINAHL, Cochrane, and clinical trial registries, and references of included studies from inception until 31^st^ July 2024. We included randomised control trials (RCTs), cluster RCTs, and associated process evaluation and cost effectiveness studies. The risk of bias was assessed using the Cochrane Risk of Bias Tool v2. PROSPERO: CRD42022315701. The study found 5527 citations, 427 full texts were screened and 149 studies (total population sample>432567) from 31 countries were included. Most studies were on tasks shared with nurses (n=83) and community health workers (n=65). Most studies focussed on cardiovascular disease (n=47), mental health (n=48), diabetes (n=27), cancer (n=20), and respiratory diseases (n=10). Seventeen studies included two or more conditions. Eighty-one percent (n=120) of studies reported at least one positive primary outcome, while 19 studies reported neutral results, one reported a negative result, eight (5.4%) reported mixed positive and neutral results, and one reported neutral and negative findings. Economic analyses indicated that task-sharing reduced total healthcare costs. Task-sharing is an effective intervention for NCDs in LMICs. It is essential to enhance the competencies and training of NPHWs, provide resources to augment their capabilities, and formalise their role in the health system and community. Optimising task-sharing for NCDs requires a holistic approach that strengthens health systems while supporting NPHWs in effectively addressing the diverse needs of their communities.

**Registration:** PROSPERO CRD42022315701.

## Background

Low and middle-income countries (LMICs, as defined by the World Bank) have a rising prevalence of non-communicable diseases (NCDs)[1,2]. They have a proportionately younger population, and yet their age-standardised mortality rate for cardiovascular diseases (CVD) is greater than that of higher-income nations[1]. People living with NCDs rely on health systems to deliver a continuum of appropriate, affordable, and high-quality services for preventing, treating, and rehabilitating NCDs. This global trend necessitates that health services transition towards models of care that are patient centred, accessible to communities, and which improve health outcomes[3]. The World Health Organization (WHO) has committed to strengthen and orient health systems to address NCDs through integrated people-centred primary health care, towards achieving universal health coverage (UHC)[4]. A set of cost-effective interventions are further recommended for wide implementation to assist countries in reaching global targets for NCDs[5].

Health workers are pivotal for NCD service delivery, yet often remain the limiting factor in health systems due to shortages or lack of training[6,7]. In order to meet UHC targets, the world needs more than 43 million additional health workers. Estimates suggest that per 10,000 population, countries need at least 20.7 physicians, 70.6 nurses and midwives, 8.2 dental personnel, and 9.4 pharmaceutical workers to achieve an effective coverage index score of 80 out of 100 [8]. The most acute health workforce shortages are experienced in LMICs, particularly in sub-Saharan Africa, South Asia, North Africa and the Middle East[8]. LMICs are faced with critical decisions on how to “shape” the health workforce to be fit-for-purpose, ensuring that future and current health workers have the required competencies, supervision, resources, and motivation to deliver quality care. An emerging approach for addressing this workforce need is ‘task sharing,’ which comprises the redistribution of health care tasks within workforces and communities[9]. According to Orkin et al., this occurs “when tasks are completed collaboratively between providers with different levels of training”[9].

The current evidence on which occupational groups can perform which tasks is limited[10]. Occupational groups with a shorter duration of pre-service education (i.e. community health workers (CHWs), non-physician clinicians, etc.) have seen a continual expansion of their tasks, based on population needs, yet their roles sometimes lack clear definition. Evidence indicates that non-physician health workers (NPHWs) (e.g. community health workers, nurses) can deliver various aspects of healthcare traditionally considered to require a physician. Although, this comes with inadequate regulatory protection, supervision, guidance, training, etc. [11]. A 2019 overview of systematic reviews analysed the barriers and facilitators to the delivery of care for NCDs by NPHWs in LMICs and provided high-level recommendations for health systems considering the adoption of task-sharing approaches [11]. However, being an overview of reviews, this study did not inspect individual interventions to identify their models of care or understand how tools and mechanisms were used to enable task-sharing. Therefore, this systematic review aims to understand the effectiveness of task-sharing and how it is used to improve NCD prevention and control in LMICs. We also explore the barriers, facilitators, and unexpected consequences of task-sharing.

## Methods

This systematic review assessed the task sharing for NCDs in LMICs by NPHWs. PROSPERO CRD42022315701.https://www.crd.york.ac.uk/prospero/display_record.php?ID=CRD42022315701

### Search strategy and selection criteria

We search MEDLINE, EMBASE, Cochrane, CENTRAL (Cochrane Central Register of Controlled Trials) and CINAHL in two phases; initially from the beginning of each database until 4th March 2022[12] and then updated the search from 1st March 2022 to 31st July 2024. Further studies were obtained from scanning reference lists of relevant studies and citation searching of key papers identified for inclusion. We searched references obtained from Cochrane Database of Systematic Reviews and search trial databases such as Clinicaltrials.gov for relevant studies. A search strategy was developed with the support of a medical librarian. We used Covidence to conduct the review[13]. Search terms are included in S1 Appendix. The following outcomes were assessed:

1. Which interventions related to prevention and control of NCDs (including prevention, promotion, management, rehabilitation, and palliation) are delivered by non-physician health workers? This included patient related outcomes (e.g., blood pressure control for hypertension related studies) indicating effectiveness of intervention delivery. We also reviewed system-related outcomes (e.g., NPHW workload) and unintended consequences of task-sharing (e.g. any harm caused).
2. Enablers and barriers for task-sharing for NCD prevention and control

Task-sharing refers to the redistribution of healthcare tasks across providers with varying levels of training to address workforce shortages. This can involve expanding the roles of existing health workers, such as nurses or CHWs or incorporating additional resources like volunteers or faith healers. The approach often utilizes a multidisciplinary team, which may include CHWs, nurses, and, in some cases, physicians [9].

Inclusion criteria comprised health facilities and communities in LMICs. Interventions involved NPHWs delivering prevention, screening, management, referral, rehabilitation, palliation for NCDs (such as diabetes, CVD, respiratory diseases, mental health disorders, cancer). Studies were included if physicians were involved with NPHWs as part of a multidisciplinary team.

This systematic review included randomised control trials (RCTs), cluster RCTs, and their associated process evaluation and cost-effectiveness studies. We included studies published in English, French and Spanish. Articles were excluded if they were not a peer reviewed article, not a report based on empirical research, pilot studies, not reported in English, Spanish or French, and research conducted on non-human subjects. Additionally, studies with fewer than 50 participants were excluded based on the sample size criterion. In both phases of search, two researchers independently reviewed and selected studies and articles against the inclusion criteria. Discrepancies between the reviewers were resolved by consultation with the team led by a third reviewer. In the case of duplicate reports, the paper with the most information was included.

### Data management and analysis

The shortlisted articles were exported to Endnote X9 (Thomson Reuters, NY, USA) for storage of study records, abstracts, and full text articles [14]. Data was stored on a password protected server-based platform that was accessible by the reviewers. Covidence, a systematic review platform, was used to streamline the process of reviewing articles. Data were collected using a standardised data extraction form. The form was piloted and optimised by two reviewers using a subset of three randomly selected studies that satisfied the eligibility criteria. Information outlined in the standardised data extraction form was collected by two reviewers independently. Data was cleaned and analysed using narrative synthesis. This was supplemented with tables and figures where appropriate. We used the PRISMA guidelines to optimize the quality of reporting.

### Risk of bias assessment

The risk of bias was assessed by two independent reviewers using the Cochrane Risk of Bias Tool v2[15, 16]. The assessment was performed at study level and focused on selection, performance, detection, attrition, and reporting bias. We did not exclude studies with a high risk of bias as we wanted to include all contexts. Furthermore, it is known that adhering to all critical aspects of study design is not always feasible in the health system setting, making some trials more vulnerable to bias[17].

### Role of funding source

The funder of the study had no role in study design, data collection, data analysis or data interpretation.

## Results

### Search and study selection

**Fig 1:**
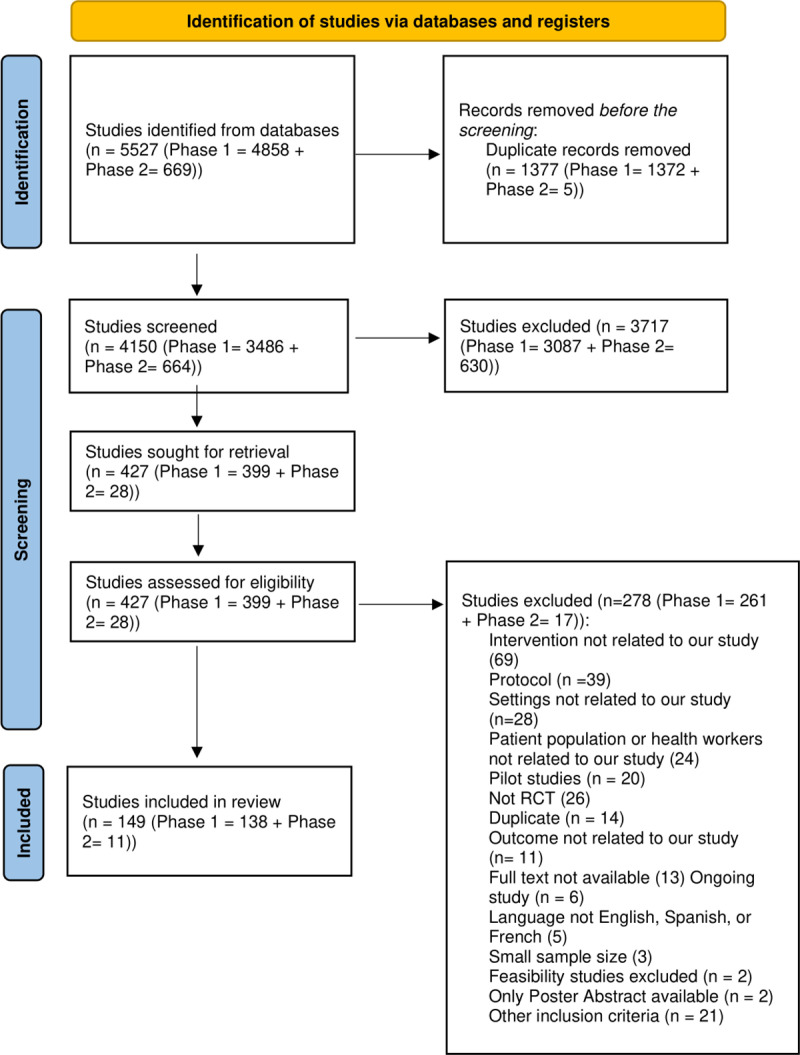
PRISMA Flow chart of study retrieval and selection.

The search retrieved 4858 potentially relevant studies in the first phase and 669 articles in the second phase, totalling 5527 studies. In the first phase, 1372 duplicates were removed, and five duplicates were removed in the second phase. After an initial screening of title and abstract of 4150 articles (3486 citations (phase 1) and 664 (phase 2)), 427 (399 (phase 1) plus 28 (phase 2)) full text articles were assessed of which 278 (261 (phase 1) plus 17 (phase 2)) did not meet the eligibility criteria. A total of 149 (138 (phase 1) and 11 (phase 2)) studies were included in the final review (Fig 1).

### Summary of included studies

One-hundred and forty-nine (149) RCTs representing at least a total of 432,567 patients were included in this review. This is because one study was a cluster randomised controlled trial with the intervention implemented and assessed at household level (29000 households). This current study did not impute the average number of individuals in each household but instead used a ratio of 1:1 for each household and individuals to get the absolute minimum sample size. The smallest study included 50 participants[18] and the two largest studies included 151,538 participants from the same cohort, which we counted once[19, 20]. The third largest studies [21, 22] included 33,995 participants from the same cohort, which we also counted once. Table 1 summarises the characteristics of included studies. Trials were published between 2001[23] and 2023[24–28] in peer reviewed journals. On average, about 10 studies were published each year since 2014-2022, with the highest being 20 published in 2020. Almost two-thirds of the studies were conducted in Asia (64%, 96/149), with 21% (31/149) from Africa, and the rest from Europe (5%, 7/149), South America (9%, 14/149), and 1% (1/149) in each of Oceania and North America. One multi-centre study was done in both Asia and South America[29].

**Table 1:** Characteristics of articles.

Author, Year	Country	Setting	Disease or condition	Participant number	Workforce included	Intervention impact^*^
Adewuya AO, et al.[38] 2019	Nigeria	Rural; Urban	Mental Health	907	Nurse; Midwife; CHW; Pharmacy technicians	Positive
Adeyemo A, et al.[87] 2013	Nigeria	Rural; Urban	CVD	668	Nurse + Doctor	Neutral
Ali M, et al.[39] 2020	India	Urban	NCD combination	404	CHW + Care coordinator, psychiatrist, diabetologist	Positive
Al Ksir K, et al.[100] 2022	Tunisia	Urban	Diabetes	66	Nurse	Positive
Arjunan P, et al. [101] 2021	India	Rural; Urban	CVD	200	Nurse	Positive
Arrossi S, et al.[102] 2015	Argentina	Rural; Urban	Cancer	6013	CHW	Positive
Azami G, et al. [103] 2018	Iran	Urban	Diabetes	142	Nurse	Positive
Bass J, et al. [104], 2016	Iran	Rural	Mental Health	209	CHW	Positive
Beratarrechea A, et al. [49], 2019	Argentina	Urban	CVD	755	CHW	Positive
Bliznashka L, et al.[105] 2021	Tanzania	Rural; Urban	Mental Health	593	CHW	Positive
Bolton P, et al.[106] 2014	Iraq	Rural	Mental Health	281	CHW	Positive
Buttorff C, et al.[40] 2012	India	Rural; Urban	Mental Health	2,796	CHW + PHC doctors + Psychiatrist	Positive
Cajanding RJ, et al.[107] 2016	Philippines	Unknown	NCD combination	123	Nurse	Positive
Cajanding RJ, et al. [108] 2017	Philippines	Urban	CVD	199	Nurse	Positive
Cakir H, et al.[88] 2006	Turkey	Urban	CVD	70	Nurse	Positive
Cal A, et al.[109] 2020	Turkey	Urban	Cancer	200	Nurse	Positive
Cappuccio FP, et al.[110] 2006	Ghana	Rural	CVD	1013	CHW	Positive
Castle P, et al.[111] 2019	Brazil	Urban	Cancer	483	CHW	Positive
Catley D, et al.[112] 2022	South Africa	Urban	Diabetes	494	CHW	Neutral
Chang Z, et al.[89] 2020	China	Rural	CVD	80	Nurse	Positive
Chao J, et al.[90] 2012	China	Urban	CVD	2400	CHW	Positive
Chaowanee,L et al.[113] 2018	Thailand	Rural	Mental Health	60	Nurse	Positive
Chatterjee S, et al.[67] 2014	India	Rural; Urban	Mental Health	282	CHW	Positive
Chen S, et al.[41] 2015	China	Urban	Mental Health	326	Nurse + doctor + psychiatrist	Positive
Chibanda D, et al.[114, 115] 2016	Zimbabwe	Rural; Urban	Mental Health	573	CHW	Positive
Dehghan N et al.[116], 2020	Iran	Urban	Diabetes	52	Nurse	Positive
DePue J, et al.[117] 2013	American Samoa	Urban	Diabetes	268	Nurse + CHW	Positive
de Souza E, et al.[118] 2014	Brazil	Urban	CVD	252	Nurse	Positive
de Souza CF, et al.[119] 2017	Brazil	Urban	Diabetes	118	CHW	Neutral
Dhoj Shrestha A, et al.[120] 2022	Nepal	Urban	Cancer	690	Female community health volunteer	Positive
Dorsey S, et al. [30], 2020	Tanzania and Kenya	Rural; Urban	Mental Health	640	Lay counsellors	Positive
Esmaeilpour-BandBoni M, et al. [45], 2021	Iran	Urban	Diabetes	66	Nurse	Positive
Fairall L, et al.[91] 2016	South Africa	Rural	NCD combination	4,393	Nurse	Neutral & negative
Gamage DG, et al.[121] 2020	India	Rural	CVD	1736	CHW	Positive
Gao G, et al.[122] 2020	China	Urban	Respiratory	180	Nurse	Positive
Garcia-Pena C, et al. [23], 2001	Mexico	Urban	CVD	718	Nurse	Positive
Gaudel P, et al.[123] 2021	Nepal	Urban	CVD	224	Nurse	Positive
George C, et al.[124] 2020	India	Rural	Mental Health	214	CHW	Positive
Getachew S, et al.[125] 2022	Ethiopia	Rural	Cancer	162	Nurse	Positive
Ghanbari E, et al. [46] 2021	Iran	Urban	Cancer	82	Nurse	Positive
Ginsburg O, et al. [50] 2014	Bangladesh	Rural	Cancer	22,337	CHW	Positive
Goudge J, et al.[76] 2018	South Africa	Rural	NCD combination	2508	Lay Health Workers	Neutral
Guerra-Riccio G, et al.[126] 2004	Brazil	Urban	CVD	100	Nurse + Pharmacist	Positive
Gul A, et al.[127] 2004	Pakistan	Urban	Mental Health	366	Community counsellors + Doctor + psychiatrist + sociologist + clinical psychologists	Positive
Gureje O, et al.[72] 2019	Nigeria	Rural; Urban	Mental Health	1178	CHW + PHC doctor	Neutral
Gureje O, et al. [31] 2020	Nigeria and Ghana	Urban	Mental Health	307	PHC providers + Traditional and faith healers	Positive
Gyawali B et al.[84] 2021	Nepal	Urban	Diabetes	244	CHW	Positive
Hanlon C, et al. [28] 2022	Ethiopia	Rural	Mental Health	329	CHW + PHC doctor	Neutral
Hasandokht T, et al.[128] 2015	Iran	Urban	CVD	161	Nurse	Positive
He J, et al.[56] 2017	Argentina	Urban	CVD	1432	CHW + PHC doctor	Positive
He J, et al.[21] 2023	China	Rural	CVD	33995^β^	Non-physician community health-care providers	Positive
Huang Y-J, et al.[129] 2017	China	Urban	NCD combination	120	Nurse	Positive
Jafar TH, et al.[74] 2009	Pakistan	Urban	CVD	1341	CHW + PHC doctor	Positive
Jafar TH et al.[130] 2010	Pakistan	Urban	CVD	4023	CHW	Positive
Jafar TH, et al. [34] 2020	Bangladesh, Pakistan, and Sri Lanka	Rural	CVD	2645	CHW	Positive
Jain V, et al.[131] 2018	India	Rural	Diabetes	299	CHW	Neutral
Jayasuriya R et al.[132] 2015	Sri Lanka	Urban	Diabetes	85	Nurse	Positive
Jeemon P, et al.[133]2021	India	Urban	CVD	1671	Nurse + CHW	Positive
Jiamjariyapon T, et al.[42] 2017	Thailand	Rural	CKD	442	Nurse, CHW, Doctor, chronic care nurse, pharmacist, nutritionist, physical therapist, community network teams	Positive
Jiang X, et al.[134] 2007	Turkey	Urban	Respiratory	61	Nurse	Positive & neutral
Jiang W, et al.[135] 2020	China	Urban	CVD	144	Nurse	Positive & neutral
Joshi R, et al.[136–138] 2012	India	Rural	CVD	1137	CHW	Positive
Joshi R, et al. [139] 2019	India	Rural	CVD	3261	CHW	Neutral
Kaaya SF, et al. [92] 2013	Tanzania	Urban	Mental Health	311	Nurse + Midwife	Positive & neutral
Kalani Z, et al.[27] 2022	Iran	Urban	CVD	80	Nurse	Positive
Kara M, et al.[140] 2007	Turkey	Urban	Respiratory	61	Nurse	Neutral
Kargar JM et al.[85] 2015	Iran	Urban	Mental Health	60	Nurse	Positive
Keliat B, et al.[141] 2020	Indonesia	Urban	Mental Health	193	Nurse	Positive
Khetan A et al.[142] 2019	India	Urban	NCD combination	1242	CHW	Positive
Khezri E, et al. [26] 2022	Iran	Urban	Cancer	72	Nurse	Positive
Kondal D, et al.[143] 2022	India	Rural	CVD	13813	CHW (ASHA)	Negative
Labhardt ND, et al.[93] 2011	Cameroon	Rural	NCD combination	221	Nurse	Positive
Li P, et al.[144], 2015	China	Urban	Respiratory	68	Nurse	Positive
Li X, et al.[47], 2020	China	Urban	Respiratory	70	Nurse	Positive
Li C, et al.[145] 2023	China	Urban	Cancer	178	Nurse	Positive
Liang R, et al.[146] 2012	China	Rural; Urban	Diabetes	62	Nurse	Positive
Liu CY, et al.[147] 2010	China	Rural; Urban	Cancer	1510	Nurse	Positive
Liu H, et al.[35] 2015	China	Urban	Diabetes	128	Dietitian	Neutral
Lund C, et al.[68] 2020	South Africa	Urban	Mental Health	425	CHW	Neutral
Ma C, et al.[148] 2014	China	Urban	CVD	120	Nurse	Positive
Malakouti SK, et al. [86], 2016	Iran	Unknown	Mental Health	241	CHW	Positive
Mash RJ, et al.[149] 2014	South Africa	Urban	Diabetes	1570	CHW	Neutral
Mehralian H, et al. [150], 2014	Iran	Unknown	CVD	110	Nurse	Positive
Mini GK, et al. [25], 2022	India	Urban	CVD	402	Nurse	Positive
Mittra I, et al.[151] 2021	India	Urban	Cancer	151538 ^π^	CHW	Positive
Mollaoĝlu M, et al.[18] 2009	Turkey	Urban	Diabetes	50	Nurse	Positive
Moreira R, et al.[152] 2015	Brazil	Rural	Diabetes	80	Nurse	Positive
Muchiri JW, et al.[36] 2016	South Africa	Rural	Diabetes	82	Dietitian	Neutral
Myers B, et al.[153] 2022	South Africa	Urban; Rural	Mental Health	1340	CHW	Positive
Neupane D, et al.[154] 2018	Nepal	Urban	CVD	1638	CHW	Positive
Ogedegbe G, et al.[155, 156] 2018	Ghana	Urban	CVD	757	Nurse	Positive
Okube OT, et al.[157] 2023	Kenya	Urban	CVD	352	Nurse	Positive
Osborn TL, et al. [158], 2021	Kenya	Unknown	Mental Health	413	High school graduates	Positive
Pace L, et al.[159] 2018	Rwanda	Rural	Cancer	1801	Nurse + CHW	Positive
Pan Y et al.[160] 2019	China	Unknown	Mental Health	112	Nurse	Positive
Patel V, et al.[43, 161] 2010	India	Rural; Urban	Mental Health	2796	CHW + PHC doctors + Psychiatrist	Positive
Peiris D, et al. [54] 2019	India	Rural	NCD combination	8,642	CHW + PHC doctor	Positive
Petersen I, et al.[162] 2021	South Africa	Urban	NCD combination	1043	Nurse + Lay counsellors	Positive
Pisani P, et al.[163] 2006	Philippines	Urban	Cancer	1293	Nurse	Neutral
Prabhakaran D, et al. [53] 2018	India	Rural	NCD combination	3698	Nurse + Doctor	Neutral
Pradeep J, et al.[164] 2014	India	Rural	Mental Health	260	CHW	Positive & neutral
Rahman A, et al. [165] 2008	Pakistan	Rural	Mental Health	903	Lady Health Workers	Positive
Rahman A, et al. [166] 2016	Pakistan	Unknown	Mental Health	346	CHW	Positive
Rahul A, et al.[167] 2021	India	Rural; Urban	Diabetes	132	Nurse	Positive
Rylance S, et al.[168] 2021	Malawi	Urban	Respiratory	120	CHW	Positive
Saffi M, et al.[169] 2014	Brazil	Urban	CVD	80	Nurse	Positive
Safren SA, et al. [170] 2021	South Africa	Unknown	Mental Health	163	Nurse	Positive
Saisanan Na Ayudhaya W, et al.[171] 2020	Thailand	Rural	Mental Health	82	Nurse + CHW	Positive
Salimzadeh H, et al.[172,173] 2018	Iran	Unknown	Cancer	122	Nurse	Positive
Samonnan T, et al.[174] 2018	Thailand	Unknown	NCD combination	93	Nurse + Researcher	Positive
Sankaranarayanan R, et al.[175] 2007	India	Rural	Cancer	80269	Nurse + CHW + Doctor	Positive
Sartorelli D, et al.[37] 2005	Brazil	Urban	Diabetes	104	Nutritionist	Positive
Scain SF, et al.[176] 2009	Brazil	Rural	Diabetes	104	Nurse	Positive
Scazufca M, et al.[177] 2022	Brazil	Urban	Mental Health	715	CHW	Positive
Schwalm JD, et al.[29] 2019	Colombia; Malaysia	Rural; Urban	CVD	1371	CHW	Positive
Secginli S, et al.[178] 2011	Turkey	Urban	Cancer	216	Nurse	Positive & neutral
Selvaraj F, et al.[179] 2012	Malaysia	Rural	CVD	297	Nurse	Positive
Sharma KK, et al. [180], 2016	India	Unknown	CVD	100	CHW	Positive
Shastri SS, et al.[20] 2014	India	Urban	Cancer	151538^π^	Primary Health care workers	Positive
Shelley D, et al.[181] 2021	Vietnam	Unknown	CVD	1312	CHW	Positive
Shi Y, et al.[182] 2020	China	Unknown	NCD combination	100	Nurse	Positive
Siabani S, et al.[183] 2016	Iran	Urban	CVD	231	Doctor + community health volunteer	Positive
Sinha B, et al.[184] 2021	India	Rural; Urban	Mental Health	1950	Trained study workers	Positive
Steffen PLS et al.[185] 2021	Brazil	Unknown	NCD combination	189	Nurse	Positive
Subramanian SC, et al.[186] 2020	India	Urban	Diabetes	70	Nurse	Neutral
Sun J, et al.[187] 2008	China	Urban	Diabetes	150	Nutritionist	Positive
Sun Y, et al.[22] 2022	China	Rural	CVD	33995 ^β^	Village doctor	Positive
Temucin E, et al.[188] 2018	Turkey	Unknown	Cancer	110	Nurse	Positive
Thakur D, et al.[189] 2019	India	Urban	NCD combination	80	Nurse	Positive
Tian M, et al.[33] 2015	China, India	Rural	CVD	2,086	CHW	Positive
Tomlinson M, et al.[190] 2018	South Africa	Urban	Mental Health	1238	CHW	Neutral
Vedanthan R, et al.[51] 2019	Kenya	Rural	CVD	1460	CHW	Positive
Wagner G et al.[191] 2016	Uganda	Urban	NCD combination	1252	Nurse	Positive
Wang Y, et al.[192] 2014	China	Urban	Respiratory	92	Nurse	Positive
Wang LH, et al.[193] 2020	China	Unknown	Respiratory	154	Nurse	Positive
Wang G, et al.[194] 2021	China	Urban	NCD combination	168	Nurse	Positive
Weiss WM, et al.[195] 2015	Iraq	Unknown	Mental Health	149	CHW	Positive
Wroe EB, et al.[196] 2021	Malawi	Rural	NCD combination	>29000^α^	CHW	Positive & Neutral
Xavier D, et al.[197] 2016	India	Urban	CVD	806	CHW	Positive
Xu DR, et al.[52] 2019	China	Rural	Mental Health	278	Lay Health Workers	Positive
Xueyu L, et al.[198] 2015	China	Urban	CVD	77	Advanced practice nurse	Positive & neutral
Yan H, et al.[44] 2021	China	Urban	CVD	249	Nurse + cardiac nurse, cardiologist, electrophysiologist, psychologist, physiotherapist	Positive & neutral
Yin Z, et al.[199] 2018	China	Rural	Diabetes	184	CHW	Neutral
You J, et al.[200] 2020	China		CVD	152	Nurse	Neutral
Yuan X, et al.[201] 2015	China	Rural	Respiratory	1008	Nurse	Positive
Pan Y, et al.[202] 2019	China		Mental Health	112	Nurse	Positive
Zhang P, et al.[203] 2017	China	Urban	CVD	236	Nurse	Positive
Zheng X, et al.[204] 2020	China		CVD	173	Nurse	Positive
Zhu X, et al.[48] 2018	China	Urban	CVD	134	Nurse	Positive

CHW: community health workers; CVD: cardiovascular disease; PHC: Primary Health Care; ^α^Cluster Randomised Controlled Trial at household level, where 29000 households were involved.

* Intervention impact relates to the primary outcome result when comparing intervention to the control; ^β^Articles used same sample; ^π^Articles used same sample.

**Fig 2:**
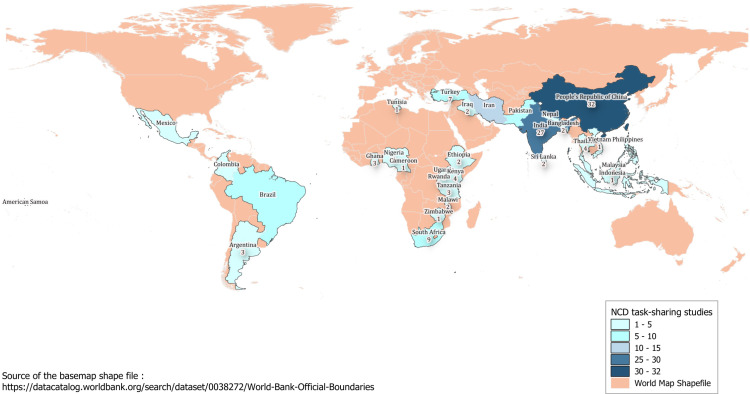
Number of studies extracted in each country. Source of the basemap shape file: https://datacatalog.worldbank.org/search/dataset/0038272/World-Bank-Official-Boundaries.

The studies originated from a total of 31 countries and there were five multi-country studies[29–33](Fig 2). Most studies (99%, 147/149) were in English and, one each in Spanish and in French. Fifty percent (75/149) of studies were conducted in urban compared to 26% (38/149) in rural areas, 17 studies (11%) were conducted in both urban and rural areas. The remaining, 13% (19/149) did not specify where they were conducted. Most studies (54%, 80/149) were conducted in the community, and 44% (66/149) were conducted in health centres or hospitals, with five studies in a combination of these settings[29–31,33,34]. Thirteen studies had a published process evaluation, and 11 studies conducted a cost-effectiveness evaluation. Workforce related outcomes (e.g. workload, frustration, satisfaction) were reported by studies that included process evaluations.

### Workforce involvement in task-sharing interventions

The workforce varied across different studies and contexts with 44% (65/149) interventions employing CHWs, and 56% (83/149) of studies using nurses (Table 2). Four studies included only dietitians or nutritionists [35–37] and three study lay health workers. Interventions included a range of services relating to prevention and health promotion (10%), screening (3%), management (36%), rehabilitation (3%) and palliation (1%). Majority (47%) involved a combination of activities including prevention, screening, management, referral and rehabilitation.

**Table 2:** Characteristics and outcomes of the interventions.

Author, Year	Disease	Activity	Management details for MLHP	Workforce	Primary outcome	Primary outcome result
Adewuya AO, et al.[38] 2019	Mental health: depression	Management, Promotion, Rehabilitation	CHW: provide enhanced Psychoeducation, including symptom education, an association between depression with interpersonal difficulties. Nurse: deliver problem-solving therapy Doctor: initiate and maintain anti-depressants medication Clinical support and supervision from the mental health team*	Doctor, nurse, CHW	Recovery score	Recovery rate in the intervention vs control group - 60.3% vs 18.2% (ARR 3.10, 95% CI 2.15 - 3.87)
Adeyemo A, et al.[87] 2013	CVD: Hypertension	Management, Promotion	Nurse: facilitation of clinic visits and health education. Treatment was implemented by a nurse with physician backup (for dose titration).	Nurse, Doctor	BP lowering medication adherence	Overall, ~77% of participants took > 98% of prescribed pills. Adherence did not differ by treatment arm
Al Ksir K, et al.[100] 2022	Diabetes	Management	Nurse-structured motivational interviewing to support youth with type-1 diabetes on their personal development and on self-management skills for their condition. Also empowered them with skills for behaviour change.	Nurse	Changes in TRAQ (Transition Readiness Assessment Questionnaire) scores	Significant differences in the mean TRAQ scores (3-months: 3.53 (sd=0.56) vs 2.11 (sd=0.57); 6-months: 4.25 (sd=0.38) vs 2.31 (sd=0.50); p<0.001) in the intervention group compared to the control group.
Ali M, et al.[39] 2020	Diabetes; Mental Health	Management, Promotion	nonphysician care coordinators (who had background on nutritional counseling, and social work): provide patient-level self-management, such as adherence to diet plans, exercise, and medications, of diabetes and depression. Diabetologist: initiate and/or modify behavioural or pharmacotherapies interventions for depression, glucose, blood pressure, and lipid management.	Nonphysician care coordinator, diabetologist	Depression symptoms and cardiometabolic indices	A significantly greater percentage of patients in the intervention group vs the usual care group met the primary outcome (71.6% vs 57.4%; risk difference, 16.9% [95% CI, 8.5%-25.2%]
Arjunan P, et al. [101] 2021	CVD: Heart Failure	Management, Promotion, Rehabilitation	Monitoring patient progress and outcomes	Nurse	Quality of life	Improvement in the physical component (t=2.23,p=.02), mental component (t= 11.17,p<.001),and disease-specific (t= 5.92,p<.001)
Arrossi S, et al.[102] 2015	Cancer: Cervical cancer	Prevention, Promotion	Distribution of cervical sample kits to CHWs. Collecting and managing data on population screening. Colposcopy and biopsy for screen-positive patients.	CHW	Screening uptake	Increase in the proportion of women having HPV test per CHW allocated to the intervention (86%) versus (20%) in the control group (p<0·0001).
Azami G, et al. [103] 2018	Diabetes	Management, Promotion	Behaviour change (motivational interview), healthy eating, being active, monitoring of blood glucose, taking medication, foot care, reducing risk, and healthy coping. Receiving telephone follow-up calls.	Nurse	HbA1c	Intervention group had significantly lower HbA1c values (47.9%) than those in the control group. At week 24, the differences increased to 62% (P<0001).
Bass J, et al. [104], 2016	Mental Health	Promotion, Management	Psychosocial support and psychoeducation, compassionate care, treatment planning, and medication management.	CHW	Depressive and dysfunction symptoms	Intervention had a significant and moderate-sized effect on depression symptoms (P= 0.02) and dysfunction (P= 0.03)
Beratarrechea A, et al. [49], 2019	CVD: CVD risk	Promotion, Screening, Prevention	mhealth tools used for screening and appointment scheduling	CHW	Attendance	49.4% of the participants with moderate or high risk of CVD completed a first visit to PHCs within 6 weeks of being screened by a CHW, compared with 13.5% in the control group.
Bliznashka L, et al.[105] 2021	Mental health	Promotion, Prevention, Promotion	Non-pharmacological therapy (e.g. developmentally appropriate stimulation of children, education on early childhood development, etc.), counselling, and promotion of caregiver responsiveness, nutrition education.	CHW	Depression and anxiety symptoms	The pooled intervention arms significantly reduced depressive symptoms and anxiety sub-scores as compared with control.
Bolton P, et al.[106] 2014	Mental health	Prevention; Management; Rehabilitation	Cognitive processing therapy (identifying, challenging and modifying maladaptive beliefs), Behavioural activation treatment for depression.	CHW	Reduction of depression symptoms	Depression effect sizes were 0.60 and 0.84 for BATD and 0.70 and 0.44 for CPT compared to all controls and BATD/CPT-specific controls respectively. Effect sizes were statistically significant
Buttorff C, et al.[40] 2012	Mental health	Screening; Management; Referral	Psychosocial care, treatment prescription, review of treatment and dosage adaptation, interpersonal therapy (relationships with others and coping strategies).	CHW	Recovery from common medical disorders	Modest evidence of an effect of the intervention on recovery (proportion recovered: 65.0% vs. 52.9% in intervention and enhanced usual care arms respectively; RR=1.22, 95%CI 1.00,1.47)
Cajanding RJ, et al.[107] 2016	Heart Failure; Mental health	Prevention; Management	Cognitive behavioural therapy (patient education, self-monitoring, skills training, cognitive restructuring and spiritual development), treatment administration.	Nurse	Quality of life, self-esteem and mood among patients living with Heart failure	After the 12-week intervention period, participants in the intervention group had significant improvement in their quality of life, self-esteem and mood scores compared with those who received only standard care.
Cajanding RJ, et al. [108] 2017	CVD: Ischaemic heart disease	Prevention; Promotion, Management	Overseeing structured discharge planning program	Nurse	Patients’ perceived functional status	Significant difference in perceived functional status scores between the control and the intervention groups of 8.59 +/- 2.29 (95%CI, 4.02-13.16; p<0.01).
Cakir H, et al.[88] 2006	CVD: Hypertension	Management	Education classes and counselling sessions	Nurse	Blood Pressure	Both SBP and DBP decreased in the intervention group but not in the control group. Mean reductions in SBP and DBP were 8.8 (SD= 5.2) and 6.9 (SD= 5.3) mmHg, respectively.
Cal A, et al.[109] 2020	Cancer: Breast cancer	Prevention; Promotion, Management	Deliver lymphedema prevention lessons	Nurse	Incidence of lymphedema	17.1% of the control group had signs of lymphedema, and none in the intervention group had lymphedema (p<.05)
Cappuccio FP, et al.[110] 2006	CVD: Hypertension	Prevention; Promotion	Health education	CHW	Urinary sodium excretion	Non-significant change in sodium excretion
Castle P, et al.[111] 2019	Cancer: Cervical Cancer	Promotion, Screening	Identifying women eligible for screening & assigning these to CHWs. Colposcopy and biopsy for screen-positive patients. Processing samples collected.	CHW	Screening uptake	Women were significantly more likely to adhere to screening when undertaking self-collection & HPV testing compared to Pap testing (p<0.001).
Catley D, et al.[112] 2022	Diabetes and CVD	Prevention	Behaviour change, strategies for setting goals on lifestyle: self-monitoring of physical activity and diet, healthy nutrition, stress management, and stimulus control.	CHW	Percentage of weight loss	Percentage of weight changes from baseline (intervention=-0.61 (95% confidence interval: -1.22 to 0.01), control: -0.44 (95% confidence interval: -1.06 to 0.18); p=) and the group differences were not statistically different (-0.17 (95% confidence interval: -1.04 to 0.71); p=71).
Chang Z, et al.[89] 2020	Multiple: Ischemic heart disease; Mental health	Management, Promotion	Percutaneous coronary intervention, counselling, cognitive behavioural therapy	Nurse	Cardiac outcomes, quality of life and mental health status	Significant increase in the scores of the 3 domains of Angina Questionnaire in the intervention group (P <.01). Mental health and physical health scores also increased (P <.01).
Chao J, et al.[90] 2012	CVD: Hypertension, Mental health	Prevention	Diet advice, psychological aspects of health, tailor-made exercise program, training on health self-management	CHW	Knowledge, psychological condition, BP monitoring, Waist-hip ratio, SBP, DBP	Improvement in (P<0.01): health knowledge score, psychological conditions, physical activity duration, BP monitoring, waist-to-hip ratio, SBP and fasting blood sugar
Chaowanee,L et al.[113] 2018	Mental health	Management, Promotion	Psychoeducation, advice to exercise.	Nurse	Mean depression scores	Mean depression scores of the intervention group were significantly low at end of study.
Chatterjee S, et al.[67] 2014	Mental health	Prevention; Promotion; Management	Education, treatment, prescription, consultations, information about illness	CHW	Reduction in symptoms and disability	Participants in the intervention group had lower scores across all subdomains of symptoms and disabilities compared to those in the control group.
Chen S, et al.[41] 2015	Mental health	Promotion; Screening; Management; Referral	Primary care nurse: consultation, treatment prescription, screening, education about illness and treatment adherence, referral Doctor: conduct depression diagnosis during screening stage, depression treatment and informed of each patient’s health questionnaire Psychiatrist: supervise and support collaborative team function	Primary care nurse, Doctor, Psychiatrist	Reduction in depression score	Patients to intervention had a significantly greater reduction in scores than did those in practices assigned to enhanced care as usual.
Chibanda D, et al.[114, 115] 2016	Mental health	Prevention; Management	Problem-solving therapy (a structured approach to identifying problems and finding workable solutions), screening, referral.	CHW	Common mental disorders	The primary outcome of SSQ-14 scores for common mental disorders was lower in the intervention group than in the control group (mean, 3.81; 95% CI, 3.28 to 4.34 vs 8.90; 95% CI,8.33 to 9.47
Dehghan N et al.[116], 2020	Diabetes	Promotion, Management, Referral	Nurse: Monitoring and evaluation of wounds, wound dressing, and blood glucose control. Coordination and management of specific cases, provision of basic care, patient education and self-care, patient referral, wound debridement, and treatment of bone abnormalities.	Nurse led team	Quality of care and HbA1C	Significant difference in quality-of-life score and HbA1c between intervention and control groups (P <.0001).
DePue J, et al.[117] 2013	Diabetes	Management	Nurse: Group education sessions, communicate with physicians about patient care needs, CHWs: appointments adherence, diabetes education, adherence to medication, problem-solving, support for self-management, referral to primary care	Nurse, CHW	HbA1c levels	At 12 months, mean HbA1c was significantly lower among participants, compared with usual care, after adjusting for confounders (b=20.53; SE = 0.21; P= 0.03).
De Souza E, et al.[118] 2014	CVD: Heart Failure	Management, Promotion	Monitoring patient progress and outcomes	Nurse	First visit to the emergency, readmission, or all-cause death	The composite endpoint of a first HF-related visit to the emergency department, hospital readmission, or all-cause death was decreased in the interventional group [relative risk 0.73; P=0.049].
De Souza CF, et al.[119] 2017	Diabetes	Management, Promotion	Diabetes education (understanding the disease, medication adherence)	CHW	HbA1c	Significant reduction in HbA1c levels in both the groups (intervention: 9.1±2.2 vs. 7.9±1.9%; control: 9.1Â2.1 vs. 8.4±2.5%, p overtime<0.001)
Dhoj Shrestha A, et al.[120] 2022	Cancer: Cervical Cancer	Screening	Health education, counselling, repeat visits, referral for visual inspection of the cervix with acetic acid.	FCHV	Change in cervical cancer screening	There was a significant increase in cervical cancer screening uptake in the intervention group compared to the control group (RR=1.48; 95% confidence interval: 1.32 to 1.66; p<0.0001)
Dorsey S, et al. [30], 2020	Mental Health	Rehabilitation	Cognitive Behavioural Therapy focusing on grief specific elements, Psychoeducation	Lay counsellors	Post traumatic stress symptoms	Compared with usual care, the intervention was more effective both after treatment and at 12 months in improving PTS in children.
Esmaeilpour-BandBoni M, et al. [45], 2021	Diabetes	Management, Promotion	Treatment adherence, Education about disease, Lifestyle education (diet, self-care and exercise)	Nurse	HbA1c	Decrease in HbA1c in the intervention group was significantly greater than that of the control group (P<0.001).
Fairall L, et al.[91] 2016	CVD: Hypertension; Diabetes; Respiratory; Mental health	Management	Educational outreach, treatment prescription, counselling, screening, review treatment/dose adjustment.	Nurse	Treatment intensification	Treatment intensification rates in intervention clinics were not superior to those in the control clinics
Gamage DG, et al.[121] 2020	CVD: Hypertension	Management, Promotion	Measurement of blood pressure, weight, waist circumference, education about diet, physical activity and hypertension	CHW	Blood pressure	1.6-fold (95%CI: 1.2-2.1; p-value=0.001) better control of hypertension in the intervention group than in the control group.
Gao G, et al.[122] 2020	Respiratory: Asthma	Promotion; Management, Prevention	Nurse: provide standard care with structured education and skills training for patients in a continuous process (outpatient sessions, home visit and telephone visit)	Nurse	Asthma control and quality of life	Significant difference (P=.002) in the asthma control score between intervention and control group. Significant difference in health-related quality of life scores between the groups.
Garcia-Pena C, et al. [23], 2001	CVD: Hypertension	Prevention; Promotion, Management	Measured blood pressure, reviewed baseline information, discussed lifestyle changes. Reviewed pharmacological treatment, provided adherence support.	Nurse	Blood pressure	36.5% of the intervention versus 6.8% of the control group had a BP of <160/90 mmHg.
Gaudel P, et al.[123] 2021	CVD: Ischaemic heart disease	Prevention; Management; Promotion	Screening eligible patients, monitoring outcomes	Nurse	Lifestyle-related risk factors	Five out of the seven studied lifestyle-related risk factors differed significantly between the study groups: diet, adherence to medication, perceived stress, and smoking and alcohol consumption.
George C, et al.[124] 2020	Mental health	Prevention; Promotion	Cognitive Behavioural Therapy, screening	CHW	Postnatal depression	30% reduced prevalence of depression in the active intervention group compared to the control groups. Not statistically significant.
Getachew S, et al.[125] 2022	Cancer: Breast cancer	Management	Health education and literacy material, empathetic counselling, phone call reminders, monitoring of medication side effects and compliance.	Nurse	Adherence to Tamoxifen	Adherence was found to be 90% (36/40) in the intervention group and 79.3% (23/29) in the control group (p=0.302).
Ghanbari E, et al. [46] 2021	Cancer: Breast cancer; Mental health	Promotion; Management	Training nurses to deliver support sessions	Nurse	Anxiety and self-esteem	Statistically significant differences between the groups 1 week after completing the intervention (P<.001).
Ginsburg O, et al. [50] 2014	Cancer: Breast cancer	Promotion; Screening; Referral	Deliver educational intervention.	CHW	Clinic attendance	Adherence was high in all three study arms. Adherence was highest for women interviewed by CHWs with smartphones and who acted as patient navigators.
Goudge J, et al.[76] 2018	CVD: Hypertension	Management, Promotion	LHWs: assist with booking appointments, retrieving and filing patient files, and providing health education (on adherence and lifestyle). Measure vital signs, assist the nurses with the prepacking of medications. Nurses: diagnoses, prescribes, and dispenses medication	Lay health workers, nurses	Blood pressure	No improvement in BP control among users of intervention clinics as compared with control clinics.
Guerra-Riccio G, et al.[126] 2004	CVD: Hypertension	Management, Promotion	Pharmacist: visited patients to deliver antihypertensive drugs and perform a pill count. Nurses: adherence support, blood pressure monitoring	Nurse, pharmacist	Blood pressure	BP declined more in intervention than in control arm (35 ± 5/19 ± 3 v 27 ± 5/9 ± 3 mm Hg).
Gul A, et al.[127] 2004	Mental health	Management	Psychotherapy/Cognitive Behavioural Therapy, Counselling.	Community based counsellors	Anxiety and/or depression	Net reduction of anxiety score in the intervention group of 21% (P=0.001) immediately after 8 weeks of counselling.
Gureje O, et al.[72] 2019	Mental health	Promotion; Management; Rehabilitation	Front-line primary care providers: psychoeducation and counselling, screening Doctor: supervision and support clinical supervisions, support, clinical emergency calls, administrative management	Front-line primary care providers (nurses, community health officers, and community health extension workers), Doctors	Remission of depression	No difference in terms of primary outcome (remission at 12 months: 76% in the intervention group vs 77% in the control group)
Gureje O, et al.[31] 2020	Mental health	Management	CHW: provide clinical support to patients, information on best clinical practices to traditional faith healers, and psychoeducation to both patients and caregivers Traditional faith healers: provide routine treatments, including herbal, ritual, and psychosocial interventions	CHW, traditional faith healers	Psychotic symptom	Participants in the intervention arm achieved a significantly better primary outcomes at 6 months than controls
Gyawali B et al.[84] 2021	Diabetes	Management, referral, Promotion	Health promotion counselling, blood glucose monitoring, referral, follow-up for medication adherence	CHW	Mean fasting blood glucose	Significantly greater reduction in intervention than control group (p<0.001)
Hanlon C, et al.[28] 2022	Mental health	Management, referral, Promotion	Nurse/psychiatric nurse/: out-patient psychiatric care CHW: psychosocial education, support people with severe disorder, follow up for people who dropped out of care Community-based lay project worker: augment engagement in care	Nurse, CHW, community-based lay project workers	Clinical psychiatric symptom severity	No evidence of inferiority of task-shared care compared with intervention. The mean score was 27.7 for task-shared care and 27.8 for control
Hasandokht T, et al.[128] 2015	CVD: Hypertension and mental health	Management	Lifestyle education (healthy dietary habits, exercise), psychosocial education	Nurse	BP (mmHg) changes	Participants had significant changes in systolic and DBP, weight, waist circumference, body mass index (BMI), energy, NaCl, and perceived stress scale
He J, et al. [56] 2017	CVD: Hypertension	Promotion; Management	CHW: health coaching, home BP monitoring, and BP audit and feedback Doctor: hypertension treatment	CHW, Doctor	Blood pressure	SBP reduction of 19.3 mm Hg (95% CI, 17.9-20.8 mm Hg) for the intervention group and 12.7 mm Hg (95% CI, 11.3-14.2 mm Hg) for the control group
He J, et al.[21] 2023	CVD: Hypertension	Management	Treatment initiation, ensure appropriate dosage for each patient, provision of discounted or free anti-hypertensive medication, health coaching on home blood pressure monitoring, medication adherence and lifestyle changes.	CHW	Myocardial infarction, stroke, heart failure requiring hospitalisation or resulting in death in a 36-month period	During a median of 36.8 months, the primary outcome was confirmed in 808 participants (1∙62% rate per year) in the intervention group and 1127 participants (2.40% rate per year) in the usual care group (hazard ratio with intervention 0∙67; 95% confidence interval: 0∙61–0∙73; p<0∙0001) and this was statistically significant.
Huang Y-J, et al.[129] 2017	CVD: Ischaemic heart disease	Prevention, Management, Promotion	Education and coaching of patients	Nurse	CHD risk factors - or do you want it to specify which ones	Compared with the control group, the intervention group had a 5 mmHg greater reduction in SBP (t = 2.01, p =.047), larger declines in glucose (t = −2.49, p =.015), cholesterol (t = −2.44, p =.017), body mass index (t = −2.58, p =.011), and depression (t = −2.05, p =.043). No significant group differences in smoking behaviour.
Jafar TH, et al.[74] 2009	CVD: Hypertension	Management; Promotion	CHWs: Family-based home health education Doctors: hypertension management	CHW, Doctor	Blood pressure	Decrease in SBP was significantly greater in the home health education and GP group (10.8 mm Hg [95% CI, 8.9 to 12.8 mm Hg]) than in the GP-only, home health education-only, or no intervention groups (5.8 mm Hg [CI, 3.9 to 7.7 mm Hg] in each; P < 0.001).
Jafar TH et al.[130] 2010	CVD: Hypertension	Management; Promotion	Health education	CHW	Blood pressure	SBP increased in the control group by 1.5mmHg and did not change appreciably (0.1mmHg) in the intervention. Difference was statistically significant (P=0.02)
Jafar TH, et al. [32, 34] 2020	CVD: Hypertension	Management; Promotion	CHWs: home visits for blood-pressure monitoring and counselling GPs: hypertension management	CHW Doctors	Blood pressure	Mean reduction in SBP was 5.2 mm Hg greater in the intervention group than in the control group (95% CI, 3.2 to 7.1; P<0.001).
Jain V, et al. [131] 2018	Diabetes	Prevention; Management	Routine fasting and post-prandial capillary glucose monitoring, Anthropometric measurements, Reinforced evidence-based prescription, lifestyle education (smoking cessation, importance of drug refilling and adherence, physical activity, dietary changes)	CHW	Fasting blood sugar, post-prandial blood sugar, glycosylated haemoglobin, lipid profile, blood pressure	No statistical difference between the intervention and the control group.
Jayasuriya R et al. [132] 2015	Diabetes	Management; Promotion	Medications log, a contract for change document, lifestyle changes (reduce total energy intake, by reducing quantity of starchy foods replaced by vegetables; physical activity promoting culturally appropriate exercise during household work and walking)	Nurse	HbA1c	Significantly lower HbA1c in intervention than control group (p=0.035)
Jeemon P, et al. [133] 2021	CVD risk factors, hypertension, diabetes	Prevention; Promotion; Screening	Health education, BP measurement, glucose measurement using POC devices, follow-up visits	CHW	Achievement or maintenance of any three: BP <140/90 mm Hg, fasting plasma glucose <110 mg/dL, low-density lipoprotein cholesterol <100 mg/dL, abstinence from tobacco	64% participants in the intervention group and 46% participants in the usual care group achieved the primary outcome. The odds of achieving the primary outcome were two times higher in the intervention group than in the usual care group (OR 2·2, 95% CI 1·7-2·7; p<0·00010)
Jiamjariyapon T, et al. [42] 2017	Other: Chronic Kidney Disease	Promotion; Management	Nurse: group counselling during hospital visit CHW: home visits to monitor compliance with the treatment.	Nurse, CHW	Mean eGFR	The mean difference of eGFR overtime in the intervention group was significantly lower than the control group by 2.74 ml/min/1.73 m2(95%CI 0.60-4.50,p= 0.009).
Jiang X, et al. [134] 2007	CVD: Ischaemic heart disease	Promotion; Management; Rehabilitation	Supervision and coaching of patients for cardiac rehabilitation	Nurse	Change of health behaviours and physiological parameters	Patients in the intervention group demonstrated a significantly better performance in walking, diet and medication adherence; a significantly greater reduction in serum lipids and significantly SBP, DBP control. The effect of the programme on smoking cessation, body weight, serum high-density lipoprotein, was not confirmed.
Jiang W, et al.[135] 2020	CVD: Ischaemic heart disease	Management; Promotion	Physician: disease assessment, drug adjustment, health education and consultation. Nurse and other team members: adaptation of the healthcare plan and outcome measurement	Nurse, doctor, dietitian, physiotherapist	Self-management behaviours	Significant difference between the intervention and control groups on the disease medical management
Joshi R, et al. [136–138] 2012	CVD: CVD risk	Prevention; Promotion; Screening; Management	CHWs: Screening of patients with CVD, education about diet, tobacco use and physical activity; recommended treatment, referral to doctor and follow up Doctor: prescribing medicines	CHW, Doctor	Proportion of high-risk individual identified	Proportion of high-risk individuals reporting that they were screened for CVD was 12% higher (intervention villages, 63.4% vs. control villages, 51.4%; p0.026)
Joshi R, et al. [139]2019	CVD: CVD risk	Prevention; Promotion	Advising dose escalation for suitable patients	CHW	Blood pressure	Significant decline in SBP (mmHg) from baseline in both groups-controls 130.3±21 to 128.3±15; intervention 130.3±21 to 127.6±15 (p<0.01 for before and after comparison) but there was no difference between the two groups at 12 months (p=0.18).
Kaaya SF, et al. [92] 2013	Multiple: Mental health; Other: HIV	Rehabilitation	Nurse/social worker/midwife: provide problem-solving therapy, psychosocial education and therapy, group counseling, education on safer sexual practices with the standard of care.	Nurse, social workers, Midwife	Depressive symptoms and increasing prenatal disclosure rates of HIV status	60% of women in the intervention group were depressed post-intervention, versus 73% in the control group [Relative Risk (RR)-0.82, 95% confidence interval (CI): 0.671.01,p-0.066]. HIV disclosure rates did not differ across the two study arms.
Kalani Z, et al. [27] 2022	Other: stroke	Prevention; promotion; Referral	Assessment of the probability of pneumonia, urinary tract infection. Planning: individualised care plan Educating the patient and family and follow up	Nurse	Incidence of pneumonia and urinary tract infection	No difference in the incidence of pneumonia between the two groups (11.6% vs. 19.2%,P= 0.35). Significant difference in the incidence of urinary tract infection (0% vs. 24.6%,P< 0.001)
Kara M, et al. [140] 2007	Respiratory: COPD	Promotion; Management	Nurse: education of the patient, provision of continuity of care	Nurse	Outcomes of patients with COPD	Statistically significant decrease in nursing diagnoses in favour of the experimental group.
Kargar JM et al.[85] 2015	Mental health	Management; Promotion	Telephone consultation, Psychosocial education and therapy.	Nurse	Depression, anxiety and stress scores	Significant differences were observed between the two groups in the post-test regarding the dimensions scores of DASS scale
Keliat B, et al.[141] 2020	Mental health	Management	Early detection, stress management, counselling therapy, family empowerment.	Nurse	Aspects of functioning (‘‘life skill’’)	Significant difference in scores before and after the implementation in the intervention group (19.94 ± 1.27 and 38.83 ± 9.32) with p <.001 and the control group (26.93 ± 12.50 and 30.89 ± 12.41) with p =.002.
Khetan A et al.[142] 2019	CVD: Hypertension	Prevention; Promotion; Screening; Management	Counselling to people with hypertension and diabetes. Encouraging physician visits, medication purchase, and medication adherence.	CHW	Blood pressure	The mean ± SD change in SPB at 2 years was −12.2 ± 19.5 mm Hg in the intervention group as compared with −6.4 ± 26.1 mm Hg in the control group. (p = 0.001).
Khezri E, et al. 2022[26]	Cancer: Breast cancer	Rehabilitation	Deliver spiritual support sessions	Nurse	Scores of hope	Mean scores of hope for the intervention and control groups were 46.71 and 40.40, respectively (P<0.05).
Kondal D, et al.[143] 2022	CVD: Hypertension	Prevention	Custom-made lifestyle modification	ASHAs	Mean change in SBP	The mean SBP increased in both the intervention and control groups at 18- months post intervention. The mean SBP increased from 124.4 mm Hg to 126.7 mm Hg and 125.7 to 126.1 mm Hg in the intervention and control group respectively. The cluster adjusted mean SBP difference was 1.91 mm Hg (95% confidence interval: -0.02 to 3.85).
Labhardt ND, et al.[93] 2011	Multiple: Hypertension; Diabetes	Management; Promotion	Counselling, understanding obstacles in long-term care and adherence support for patients	Nurse	Patient retention	Retention rates in the incentive and letter group were 60% and 65%, respectively, compared with 29% in the control group. The differences between the groups were significant (95% CI: 21% to 46%;P< 0.001).
Li P, et al. [144], 2015	Respiratory: COPD	Management; Rehabilitation	Education and guidance about use of inhalers, supporting their care plan including exercises for rehabilitation.	Nurse	Preventing acute exacerbations, improving health-related quality of life among patient with COPD	Total and subscale scores (P < 0.05) of SOLDQ and CSES significantly improved compared to the baseline ones in the intervention group. At 12 weeks, scores showed a sustained and significant growth in the intervention group (P < 0.05). Significantly lower average medical expenses than the control group (P < 0.05)
Li X, et al. [47], 2020	Respiratory: COPD	Promotion, Management	Education about COPD, diet and physical activity and follow-up with adherence support (for inhalers)	Nurse	Quality of life	Significant differences in the total quality of life score (20.29 ± 10.03 vs 30.14 ± 12.52) between the intervention group and the control group (P < .05).
Li C, et al.[145] 2023	Cancer: Gastrointestinal cancer	Prevention and Screening	Nurse-led counselling, health education, tailored information, addressing hepatocellular carcinoma screening barriers,	Nurse	Hepatocellular carcinoma screening rates after 6-months	Significant differences in the hepatocellular carcinoma screening uptake rate (75.6%vs.42.1%;χ2=17.909; p<0.001) in the intervention group compared to the control group.
Liang R, et al.[146] 2012	Diabetes	Prevention; Promotion; Management	Education on foot care, foot examination, follow-up visits	Nurse	Knowledge and foot care behaviours	At 1 and 2 years later, diabetes knowledge and foot care behaviour was significantly higher in the intervention than control group (p<0.05 or p<0.01)
Liu CY, et al.[147] 2010	Cancer: Breast cancer	Promotion; Screening	Deliver education for breast cancer examination	Nurse	Self-examination frequency	34% of the intervention, 11% of the control, group did breast self-examination (P<0.001).
Liu H, et al.[35] 2015	Diabetes	Management; Promotion	Education and management of diet	Dietitian	Anthropometric measurements	No significant change in anthropometric measures
Lund C, et al.[68] 2020	Mental health	Management	Counselling, psychoeducation, problem-solving skills, behavioural activation, healthy thinking, relaxation training, social support, screening.	CHW	Reduction in perinatal depression	There were no differences between the two arms at eight months gestation, three months, or 12 months postpartum.
Ma C, et al.[148] 2014	CVD: Hypertension	Management	Counselling and adherence support	Nurse	Blood pressure	SBP, DBP of the intervention group decreased compared with ones of the control group, the difference values were 4.92 and 2.58, respectively.
Malakouti SK, et al. [86], 2016	Mental Health	Management	Consultation, referral, review of treatment.	Nurse	Hospitalization rate	Rate of rehospitalization for the telephone follow-up and as-usual groups were respectively 1.5 and 2.5 times higher than the home-visit group
Mash RJ, et al.[149] 2014	Diabetes	Prevention	Education on disease and medication, lifestyle education	CHW	Primary outcomes were diabetes self-care activities, 5% weight loss and a 1%reduction in HbA1clevels	No significant improvement was found in outcomes, apart from a significant reduction in mean systolic (-4.65 mmHg, 95% CI 9.18 to -0.12; P=0.04) and diastolic blood pressure (-3.30 mmHg, 95% CI -5.35to -1.26; P=0.002)
Mehralian H, et al. [150], 2014	CVD: Heart Failure	Promotion	Monitoring patient progress and outcomes	Nurse	Quality of life	At hospital discharge, mean score of all 8 sub-score of SF-36 questionnaire was 63.4 in patients of intervention and 61.1 in patients of control, respectively (P> 0.05). After 6 months, mean score of QOL was higher in intervention than in control.
Mini GK, et al.[25], 2022	CVD: Hypertension	Management; Promotion	Educate schoolteachers about hypertension control, healthy lifestyle practices, and self‐management of hypertension and related NCDs and risk factors.	Nurse	Blood pressure	A greater proportion of intervention participants (49.0%) achieved hypertension control than the usual care participants (38.2%)
Mittra I, et al.[151] 2021	Breast cancer	Prevention; Screening	Deliver educational intervention	CHW	Downstaging of breast cancer at diagnosis and reduction in mortality from breast cancer	Breast cancer was detected at an earlier age in the intervention group than in the control group (age 55.18 (standard deviation 9.10) v 56.50 (9.10); P=0.01), with a significant reduction in the proportion of women with stage III or IV disease (37% (n=220) v 47% (n=271), P=0.001).
Mollaoĝlu M, et al.[18] 2009	Diabetes	Prevention	Dietary education, exercise, measuring of blood and urine glucose	Nurse	HbA1c	Intervention arm mean HbA1c values fell from 9.5 Â± 1.7 mg/dl to 7.5 Â± 1.3 mg/dl. Control group, decreased from 9.7 Â± 1.6 mg/dl to 9.6 Â± 1.6 mg/dl. The difference between the groups was statistically significant (p <0.05)
Moreira R, et al.[152] 2015	Diabetes	Management; Promotion	Create individualised care plan, Blood pressure and glucose monitoring, evaluate insulin application technique, assess proper disposal of contaminated materials, promote health education (capillary glycemia test, insulin administration, foot care).	Nurse	HbA1c	HbA1c was reduced from an average of 10.33% to 9.0% (p <.01) in the intervention group and from 9.57% to 8.93% (p =.05) in the control group; the group by time effect was not significant.
Muchiri JW, et al.[36] 2016	Diabetes	Management	education programme (health diet advice, meal planning, meal preparation), understanding diabetes	Dietitian	HbA1c	Differences in HbA1c (primary outcome) were -0·64% (P=0·15) at 6 months and -0·63% (P=0·16) at 12 months in favour of the intervention group
Myers B, et al.[153] 2022	Mental health	Management	Health promotion and adherence support for HIV and non-communicable diseases in general, mental health education, use motivational interviewing techniques to build rapport and develop readiness to change, develop a change plan	CHW	Changes in depression and alcohol use	The trial had three groups and there were significant differences in the depression scales at 6 months (-4.96; 95% confidence interval: -7.47 to -2.45; p<0.0001) between the dedicated and treatment as usual group and between the designated and treatment as usual group (-2.76; 95% confidence interval: -5.28 to -0.26; p=0.031). After 12-months the scores were also statistically different; (-5.02; 95% confidence interval: -7.51 to -2.54; p<0.0001) between the dedicated and treatment as usual group and between the designated and treatment as usual group (-6.38; 95% confidence interval: -8.89 to -3.88; p<0.0001). On Alcohol Use Disorders Identification Test (AUDIT); at 6 months there were statistically significant differences (-2.75; 95% confidence interval: -5.31 to -0.19; p=0.035) between the dedicated and treatment as usual group and between the designated and treatment as usual group (-2.80; 95% confidence interval: -5.41 to -0.19; p=0.035). After 12-months the scores were not statistically different; (-1.73; 95% confidence interval: -4.38 to 0.92; p=0.20) between the dedicated and treatment as usual group and between the designated and treatment as usual group (-1.97; 95% confidence interval: -4.65 to 0.71; p=0.15).
Neupane D, et al.[154] 2018	CVD: Hypertension	Prevention; Management; Referral	Lifestyle counselling and blood pressure monitoring	CHW	Blood pressure	Mean SBP at 1 year was significantly lower in the intervention group than in the control group for all cohorts: the difference was −2·28 mm Hg (95% CI −3·77 to −0·79, p=0·003) for participants who were normotensive, −3·08 mm Hg (–5·58 to −0·59, p=0·015) for participants who were prehypertensive, and −4·90 mm Hg (–7·78 to −2·00, p=0·001) for participants who were hypertensive
Ogedegbe G, et al.[155, 156] 2018	CVD: Hypertension	Promotion; Management	Cardiovascular risk assessment, lifestyle counselling, and initiation/titration of antihypertensive medications	Nurse	Blood pressure	Across both groups (healthcare insurance only and healthcare insurance + nurse visit), there was a significant and sustained reduction in SBP.
Okube OT, et al.[157] 2023	CVD: CVD risk	Health promotion and prevention	Individualised and group-based health education at three time points, education on major CVD behavioural risk factors, including advice on salt and sugar reduction, avoidance or reduction on processed/fast foods and saturated fats. Dietary advice on plating and correct portion sizes.	Nurse	Changes in CVD knowledge level	Significant differences in the CVD knowledge levels from baseline (74.4% vs 29.0%; p<0.0001) in the intervention group compared to the control group.
Osborn TL, et al. [158], 2021	Mental health	Prevention; Promotion; Management	Layperson-delivered group intervention on growth mindset, gratitude, and value affirmation	Lay-person	Depression and anxiety symptoms	Reductions in depressive symptoms at post-treatment (Cohen d= 0.35 [95% CI, 0.09-0.60]), and 7-month follow-up (Cohend= 0.45 [95% CI, 0.19-0.71]) Reductions in anxiety symptoms at post treatment (Cohen d= 0.37 [95% CI,0.11-0.63]), and 7-monthfollow-up (Cohen d= 0.44 [95% CI, 0.18-0.71]).
Pace L, et al.[159] 2018	Cancer: Breast cancer	Screening; Promotion; Referral	Nurse: provide screening breast concerns, and refer patients to the breast clinic CHW: provide educational sessions and encourage individual women with breast symptoms to seek prompt evaluation at health centers	Nurse and CHWs	Health care use and patient outcomes	1,500 patients sought care at intervention HCs for breast concerns versus 600 at control HCs. Biopsy rate was 36.6 per 100,000 person-years from intervention versus 8.9 per 100,000 from control arm (P <.001).
Pan Y et al. [160] 2019	Mental health	Prevention; Promotion; Management	Nurse: cognitive behavioural interventions, consultation	Nurse	Depressive symptoms and coping strategies	Significant difference in depressive symptoms and active coping between groups over time, with p<.001 for the interaction between depressive symptoms and groups and p<.01 for the interaction between active coping and groups.
Pan Y, et al.[202] 2019	Mental health	Promotion; Rehabilitation	Psycho-education, Cognitive behavioural therapy	Nurse	Depressive symptoms of caregivers of persons	Improved mutuality (p = 0.049) and active coping (p = 0.0001) and decreased passive coping (p = 0.001) were found to predict the reduction of depressive symptoms among caregiver.
Patel V, et al.[43, 161] 2010	Mental health	Management; Promotion; Referral	Lay health counsellor: deliver psychoeducation, interpersonal psychotherapy, treatment prescription and administration Physician: support and supervise the lay health counsellor, follow, and refer high risk participants Psychiatrist: assess high-risk participants and consult a primary health physician	Lay health counsellors, primary care physician and a psychiatrist (clinical specialist)	Recovery from common medical disorders	The intervention had no significant effect on recovery from common mental disorders in patients with depression.
Peiris D, et al. [54] 2019	CVD: Hypertension	Screening; Management; Referral	CHWs: screening, education, referral, follow-up, adherence support Doctor: diagnoses, initiation of treatment	CHW, Doctor	Blood pressure	No difference in the proportion of people achieving SBP targets (41.2% vs 39.2%; adjusted odds ratio (OR) 1.01 95% CI 0.76–1.35) or receiving BP-lowering medications in the intervention vs control periods respectively.
Petersen I, et al.[162] 2021	Mental health	Management; Referral; Promotion	Counselling, treatment prescription, referral	Nurse	Depressive symptoms	Retention was good and participants in the group based IPT intervention showed significant reduction in depressive symptoms on completion
Pisani P, et al.[163] 2006	Cancer: Breast cancer	Prevention; Promotion; Referral	Nurse and midwives: provide mass screening by clinical examination of the breast (CBE), educate technique of breast self-examination, and refer women with positive results. Doctor: perform needle biopsies	Nurse, Midwife, Doctor	Incidence of breast cancers	In the single screening round 92% accepted, 3,479 were detected and referred for diagnosis. Of these 35% completed diagnostic follow-up
Prabhakaran D, et al. [53] 2018	Multiple: Hypertension; Diabetes; Mental health	Management	Lifestyle education, Treatment prescription, Review treatment, consultation, blood pressure and glucose measurement.	CHW	Blood pressure and HbA1c	No difference in change in SBP and HbA1c between the 2 arms.
Pradeep J, et al.[164] 2014	Mental health	Prevention; Promotion; Referral; Management	Screening, Pill count, adherence counselling, referral, Education about disease, Monitoring side effects, prescription	CHW	Treatment seeking and completion Changes in severity of depression and changes in quality of life	Significantly greater number of women from the intervention group completed the treatment and were on treatment for a longer duration compared to the control group. No significant differences in the severity of depression or quality of life between the groups.
Rahman A, et al. [165]2008	Mental health	Promotion; Management	Consultation, Counselling, referral	Nurse	Infant nutrition	The differences in weight-for-age and height-for-age Z scores for infants in the two groups were not significant at 6 or 12 months
Rahman A, et al.[166] 2016	Mental health	Management; Rehabilitation; Promotion	Problem solving therapy, behavioural activation, strengthening social support, stress management, adherence counselling	Nurse	Anxiety and depression symptoms	After 3 months of treatment, the intervention group had significantly lower mean (SD) HADS scores than the control group for anxiety (7.25 [3.63] vs 10.03[3.87]) and depression (6.30 [3.40] vs 9.27 [3.56])
Rahul A, et al.[167] 2021	Diabetes	Management	Lifestyle education (dietary advice, encourage physical activity, cessation of smoking and alcoholism, drug compliance), treatment adherence, understanding the disease, monitoring glucose	CHW	Fasting blood sugar	mean fasting blood sugar values dropped in both groups, the drop being higher in the intervention group. The intervention was associated with a significant reduction in FBS at the end of 6-month follow-up after controlling for the effect of baseline (p<0.001)
Rylance S, et al.[168] 2021	Respiratory: COPD	Management	Nurse: Clinical assessment, optimisation of inhaled treatment Lay Educators: individualised asthma education	Lay educators, nurse	Asthma symptom control	At 3 months, children in the intervention arm had a mean (SD) cACT of 22.9 (2.3), compared with 20.8 (3.0) in stan-dard care (p<0.001). Children receiving the intervention had a greater mean (SD) change in cACT score from baseline; 2.7 (2.8) compared with 0.6 (2.8) for standard care participants, a differ-ence of 2.1 points (95%CI: 1.1 to 3.1, p<0.001)
Saffi M, et al.[169] 2014	CVD: CVD risk	Prevention; Management	Education and follow-up Develop exercise and dietary goals with patients	Nurse	10-year cardiovascular risk scores	A 1.7 point (â∘’13.6%) reduction in risk score was recorded in the intervention group, vs a 1.2 point increase in risk score (+11%) in the control group (p=0.011
Safren SA, et al.[170] 2021	Mental health	Promotion; Management; Rehabilitation	Cognitive Behavioural Therapy: adherence counselling, psychoeducation, mood monitoring, problem solving, Relaxation training, relapse prevention	Nurse	Depression and medication adherence	t 4 months, the HAMD scores in the CBT-AD condition improved by an estimated 4.88 points more, and for weekly adherence, 1.61 percentage points more per week than ETAU.
Salimzadeh H, et al.[172,173] 2018	Cancer: Gastrointestinal cancer	Prevention; Screening	Nurse: provide motivational interview counselling sessions and training	Nurse	Colonoscopy in first degree relatives within six months	At follow-up, the uptake of screening colonoscopy in the intervention group was 83.5% versus 48.2% in controls (P<.001).
Saisanan Na Ayudhaya W, et al.[171] 2020	Mental health	Management	CHW: Screening Nurse: behaviour activation aimed to increase engagement in activities and follow up patients	Nurse, CHW	Thai Geriatric Depression Scale and Depression Anxiety Stress Scales	The adjusted mean change in depression scores improved significantly in the intervention group compared to the usual care-only group, and anxiety score improved significantly at 6 months
Samonnan T, et al.[174] 2018	Mental health	Promotion; Rehabilitation	Researcher: serve as interventionist and guide on the intervention for participants to raising self-awareness, Support self-care, lifestyle education Nurse: provide routine care, including health-related education about chemotherapy	Nurse, researcher	Psychological symptom experiences	Non-significant difference in the mean scores of psychological symptom experiences between the two groups, but there was a significant time difference and a significant interaction effect.
Sankaranarayanan R, et al.[175] 2007	Cancer: Cervical cancer	Screening; Promotion; Referral	Identifying women eligible for screening. Collecting and managing data on population screening. Doctors: supervising nurses, performing LEEP	CHW	Incidence and mortality	The intervention group had lower cervical cancer incidence and mortality rates than the control group. Overall, the intervention group had a significant reduction in cervical cancer incidence and a significant reduction in cervical cancer mortality compared with the control group.
Sartorelli D, et al.[37] 2005	CVD: CVD risk	Prevention	Individualised nutritional counselling sessions	Dietitian	Cholesterol	At 6 months total cholesterol (-12.3% vs.-0.2%), low-density lipoprotein (LDL) cholesterol -15.5% vs.+4.0%) and fasting plasma glucose (-5.6% vs.+0.1%) in the intervention group compared with the control group(P<0.05).
Scain SF, et al.[176] 2009	Diabetes	Promotion, Management	Lifestyle education (self-care, healthier dietary habits, exercise, self-monitoring, foot care), understanding of diabetes and its complications.	Nurse	HbA1c	HbA1C levels decreased significantly in the intervention group after the 4th month and remained lower than in the control group until the 12th month.
Scazufca M, et al.[177].2022	Mental Health	Prevention and Management	The psychosocial intervention consisted of a 17-week programme delivered by CHWs. Through these sessions, they monitored depression symptoms, checked chronic conditions and antidepressant medication adherence (if on medication), psychoeducation about depression, simple strategies to improve mood, discuss difficult cases with the team, Behavioural activation (introduce pleasant activities in a stepped way), introduce relapse prevention strategies, inform team members about the end of the intervention and summarise case.	CHW	Depression recovery at 8-months	Significant differences in the recovery from depression at 8-months (62.5%vs.44.0%; OR=2.16 (95% confidence interval: 1.47-3.18); p<0.0001) in the intervention group compared to the control group.
Schwalm JD, et al.[29] 2019	CVD: CVD risk	Promotion, Screening; Management	Screening, health education, adherence support, recommended treatment, in some cases they delivered medicines to patients Doctor: prescribed medicines	Nurse	CVD risk	Statistically significant reduction in Framingham Risk Score for 10-year CVD risk
Secginli S, et al.[178] 2011	Cancer: Breast cancer	Promotion, Screening	Developing and distributing educational materials.	Nurse	Screening	The breast health promotion program significantly increased breast self-examination frequency. No significant differences existed in mammography and clinical breast examination rates between the two groups at 6 months.
Selvaraj F, et al.[179] 2012	CVD: CVD risk	Management; Promotion	Provided self-management support and patient empowerment, reinforcement of the health education information and adherence support	Nurse	LDL-C	LDL-C were 30.09% and 27.54% for the intervention group and control groups, respectively. The difference of mean change in the intervention group was 2.55% lower than the control group(p=0.288).
Sharma KK, et al. [180], 2016	CVD: Other heart disease	Promotion, Management	Follow-up care	CHW	Medication adherence	At 12 and 24 months, respectively, in intervention vs control groups adherence (>80%)
Shastri SS, et al.[20] 2014	Cancer: Cervical cancer	Promotion, Screening	Identifying women eligible for screening. Collecting and managing data on population screening. Doctors: supervising PHWs Community rapport-building procedures	CHW	Mortality.	The screening group showed a statistically significant 31% reduction in cervical cancer mortality (RR = 0.69; 95% CI = 0.54 to 0.88; P =.003)
Shelley D, et al.[181] 2021	Other: Tobacco use	Prevention, Promotion, Management	PHC workers: Used the 4As to educate, support and manage patients: Ask about tobacco use, Advise to quit, Assess readiness, Assist with brief counselling CHW: intensive counselling for tobacco cessation	PHC providers, CHWs	Adoption of the 4As or 4As + intensive counselling	Adoption of the 4As increased significantly across both study arms (all p <.001).
Shi Y, et al.[182] 2020	Multiple: Cancer: Cervical cancer; Mental health	Promotion, Rehabilitation	Referral, Sexual psychological rehabilitation, counselling, sexual yoga and pelvic floor rehabilitation exercises, Sex education	Nurse	Sexual Function	Compared with participants in the control group, participants in the intervention group showed significant improvements in sexual function and improvements in their levels of depression and well-being
Siabani S, et al.[183] 2016	CVD: Heart Failure	Management, Promotion	Community health volunteer: provide home-based face-to-face self-care education for patients with chronic heart failure Nurse and a general practitioner: provide education in the hospital	Community health volunteers(CHV), Nurse, General practitioner	Self-care components	After intervention, self-care components were significantly increased in all three groups, with the exception of self-care confidence, which did not change significantly in the usual care group. Self-care scales in patients educated by CHVs improved to a similar extent to patients educated by nurse and general practitioners.
Sinha B, et al.[184] 2021	Mental health	Prevention; Promotion	Consultation, breastfeeding counselling, referral	Nurse	Depressive symptoms	The proportion of mothers with moderate-to-severe postpartum depressive symptoms was 10.8% (95% CI, 8.9%-12.9%; 105 of 974 mothers) in the intervention group vs 13.6% (95% CI, 11.4%-16.1%; 116 of 852 mothers) in the control group
Steffen PLS et al.[185] 2021	Multiple: Hypertension; Diabetes	Management	Motivational interviewing	Nurse	HbA1c	0.4% (p<0.01) reduction in HbA1c levels for the intervention group with a statistical significance and a small effect size (0.3)
Subramanian SC, et al.[186] 2020	Diabetes	Management; Promotion	Prescription, teaching on diabetes and its complications, education on treatment adherence, lifestyle education (self-management, exercise, healthier dietary habits)	Nurse	Diabetes Self-Management Questionnaire	Significant increase in physical activity in intervention (2.17 to 9.02) and decrease in control (1.28 to 1.22). No significant differences in dietary control, blood glucose monitoring, medication adherence, physician contact
Sun J, et al.[187] 2008	Diabetes	Prevention; Management	Education on self-management, Lifestyle education (behavioural and lifestyle modifications including physical activity and healthier dietary habits)	Dietitian	Fasting blood glucose, insulin, systolic and diastolic blood pressures	The Intervention Group improved fasting blood glucose, insulin, systolic and diastolic blood pressures compared to Reference Group (p<0.05).
Sun Y, et al.[22] 2022	CVD: Hypertension	Management	Treatment initiation, ensure appropriate dosage for each patient, health coaching on home blood pressure monitoring, medication adherence and lifestyle changes.	CHW	Blood pressure <130/80 mm Hg at 18 months	There were statistically differences in blood pressure changes between the intervention and control group at 18-months (group difference=37.0%; 95% confidence interval=34.9 to 39.1%; p<0.0001). In the intervention group, 57.0% of patients had a blood pressure of less than 130/80 mm Hg compared to 19.9% in the control group.
Temucin E, et al.[188] 2018	Cancer: Gastrointestinal cancer	Prevention; Promotion; Screening	Screening, navigation and counselling	Nurse	Screening participation	The FOBT (82 and 84%, respectively) and colonoscopy completion rates (15 and 22%, respectively) were significantly higher in the intervention versus control groups at 3 and 6 months follow-up.
Thakur D, et al.[189] 2019	Multiple: Mental health; Other: intracranial tumours	Management; Rehabilitation; Promotion	Assessment of behavioural symptoms, counselling, diagnosis, consultation, discharge education, prevention of complications	CHW	Behavioural symptoms	Patients in the intervention group had significantly fewer behavioural symptoms and less severity of behavioural symptoms as compared to the control group.
Tian M, et al.[33] 2015	CVD: Hypertension	Promotion; Management	China: CHWs were aided by the smartphone-based app to prescribe medication and lifestyle counselling India: CHWs screened and provided counselling to patients using an app. Doctors: prescribed the medications	CHW	Adherence to anti-hypertensive medication	The intervention group had a 25.5% (P<0.001) higher net increase in the proportion of patient-reported antihypertensive medication use pre- and post-intervention.
Tomlinson M, et al.[190] 2018	Mental health	Prevention; Management; Promotion	Education on HIV disclosure, alcohol prevention education, breastfeeding education and counselling	CHW	Physical and cognitive outcomes	No significant differences
Vedanthan R, et al.[51] 2019	CVD: Hypertension	Screening; Management; Referral; Promotion	1 group of CHWs used paper-based tools to provide tailored behavioural communication; Another group of CHWs used smartphones to provide tailored behavioural communication	CHW	Impact on linkage to care	Linkage to care was 49% overall, with significantly greater linkage in the usual care (50%) and smartphone arm (54%) than paper-based arm (43%)
Wagner G et al.[191] 2016	Multiple: Mental health; Other: HIV	Management	Diagnosis of depression; initiating antidepressant therapy (using a protocol/algorithm)	Nurse	Screening	Mean number of adult clients seen each clinic day across all sites in the structured protocol arm was 69.1 (SD = 33.5; range: 35.6-97.8 across sites), which was more than the 58.0(SD = 30.6; range: 29.4-90.3) clients seen in the clinical acumen arm (t = 7.35, df = 1641, p<.0001).
Wang Y, et al.[192] 2014	Respiratory: COPD	Management; Promotion	Assist patients to understand the susceptibility and severity of COPD, understand benefits of treatment, Education for healthy behaviours, monitor their symptoms.	Nurse	Health belief and self-efficacy, degree of difficulty in breathing, activities of daily life, six-minute walking distance and pulmonary function indicators.	Patients in the intervention group had significantly higher mean total scores in the Health Belief Scale and the COPD Self-Efficacy Scale, and in all the sub scales, than in the control group except the perceived disease seriousness. Results showed that the value of FEV1/FVC ratio had a significant difference between study groups before and after the intervention.
Wang LH, et al.[193] 2020	Respiratory: COPD	Promotion; Management; Prevention	Patient assessment for physical activity self-management needs, management of COPD, follow up of patients	Nurse	COPD-related hospital admissions and emergency department visits	Compared to the control group, participants in the intervention group showed significantly fewer COPD-related hospital admissions (P = 0.03) and emergency department visits (P = 0.001)
Wang G, et al.[194] 2021	Cancer: Breast cancer	Management; Promotion	Education about the condition, dealing with relationships and planning for the future	Nurse	Post traumatic growth, anxiety and depression	Compared to control group, participants in intervention group reported higher level of post-traumatic growth (p < 0.01 or 0.05), reduced anxiety and depression (p < 0.01 or 0.05 and p < 0.01 or 0.05).
Weiss WM, et al.[195] 2015	Mental health	Management; Rehabilitation; Promotion	Counselling, Cognitive processing therapy, psychoeducation, Referral,	CHW	The Posttraumatic Growth Inventory (PTGI)	Significant improvements on PTG were observed in the intervention group compared with the control group
Wroe EB, et al.[196] 2021	Hypertension; Diabetes; Asthma; Mental health, malnutrition and TB, family planning, antenatal care	Prevention; Screening; Management; Referral	Lifestyle education, screening, Referral, Treatment adherence monitoring	CHW	Proportion of NCD clients who default from care this month	Decrease of approximately 20% in the rate of patients defaulting from chronic NCD care each month (−0.8 percentage points (pp) (95% credible interval: −2.5 to 0.5)) while maintaining the already low default rates for HIV patients (0.0 pp, 95% CI: −0.6 to 0.5).
Xavier D, et al.[197] 2016	CVD: CVD risk	Prevention; Management	Educate patients on healthy lifestyle and drugs, and measures to enhance adherence.	CHW	Adherence to medications	Adherence (≥80%) to prescribed evidence-based drugs was higher in the intervention group than in the control group (97% *vs* 92%, odds ratio [OR] 2·62, 95% CI 1·32–5·19; p=0·006)
Xu DR, et al.[52] 2019	Mental health	Management; Promotion; Referral	Adherence support, Observed treatment ingestion.	Lay health worker	Medication adherence	Medication adherence was 27% greater in the intervention group (0.61) than in the control group (0.48).
Xueyu L, et al.[198] 2015	CVD: CVD risk	Rehabilitation; Management; Promotion	Nurse led rehabilitation training	Nurse	Quality of life	No significant direct effects for group for SF-36. The intervention group showed improvement in physical functioning role-physical, bodily pain, and vitality (p < 0.05) on the SF-36.
Yan H, et al.[44] 2021	CVD: CVD risk	Management; Promotion	Nurse: provide usual care and nurse-led multidisciplinary team management	Nurse	Cardiovascular hospitalization and cardiovascular death.	Patients under intervention (a nurse-led multidisciplinary team approach) showed fewer cardiovascular hospitalizations (17 vs. 35,p=0.006) than those receiving usual care. No difference was found with cardiovascular death.
Yin Z, et al.[199] 2018	Diabetes	Management	Goal setting and education (diet, physical activity) of patients	Nurse	Diabetes	No significant reduction in blood glucose
You J, et al.[200] 2020	CVD: Heart Failure	Promotion; Management	Monitoring patient progress and outcomes	Nurse	Medication adherence	No significant differences
Yuan X, et al.[201] 2015	Respiratory: COPD	Prevention; Management	Health education, smoking cessation counselling, and education on management of COPD	Nurse	Incidence, decline in lung function, and mortality	COPD incidence was lower in the intervention group than in the control group (10%vs16%, P<0.05). Intervention group had a significantly lower cumulative COPD-related death rate than the control group (37%vs47%, P<0.05).
Zhang P, et al.[203] 2017	CVD: Ischaemic heart disease	Management; Promotion	Structured assessment and health education, health education and coaching via home visits and phone calls	Nurse	BP, fasting blood glucose, triglycerides, high density and low-density lipoprotein cholesterol	Clinical outcomes showed significant differences between the control and intervention groups over time.
Zheng X, et al.[204] 2020	CVD: CVD risk	Promotion; Management	Face to face education sessions with follow up via phone calls.	Nurse	Cardiovascular risk	Decreased cardiovascular risk was found in the intervention group, but no significant group-by-time effect was detected
Zhu X, et al.[48] 2018	CVD: Hypertension	Management; Referral; Promotion	Nurse: home visits, telephone follow-ups and referral when necessary. Doctor: pharmacological treatment for referred patients.	Nurse, doctor	Blood pressure	SBP reduction was about 14.72mmHg in the intervention, which was greater than that in the control group(9.22mmHg). DBP decreased more in the intervention (7.43mmHg) than in the control group (5.14mmHg).

Sd=standard deviation; SBP=systolic blood pressure; RR=Risk Ratio, Cardiovascular disease.

By comparison, CHWs were mostly involved in health education, screening and referrals. CHWs also conducted management in few studies. Some studies had multidisciplinary teams including the primary healthcare (PHC) team of CHWs, nurses, PHC doctor and a specialist or team of specialists[38–44]. Most studies reported providing training or employed a trained health workforce (69%, 103/149) and 39% (59/149) of studies reported having a supervisory structure for the workforce. None of the studies indicated evidence of harm of task-sharing NCD related interventions.

### Uses of digital health in task-sharing

The intervention involved digital health solutions in 15 studies. Four of the digital health studies included nurses[45–48] and six included CHWs[29, 33, 49–51] or lay health workers[52]. Three studies had a team of a doctor with either CHW or nurse, and one study involved a multidisciplinary team of five specialists and a nurse[44]. Twelve out of the 15 studies included management of the condition[29,33,44,48–56], one focussed on rehabilitation[57], one on screening[58], and one focussed on education[46].

Digital health interventions included the use of clinical decision support tools and electronic health records to help the NPHW with diagnosis, treatment and referral[29,33,49,51,53,54]. In one study, a smartphone application was used by the patients to set reminders to improve medicine adherence[24]. Smartphone applications were also used to train CHWs[49, 50, 54], to enable online patient support groups[46] and to correspond with patients[24,52].

### Diseases addressed by task-sharing interventions

The diseases or conditions investigated varied between the studies (Table 1). Most studies focussed on cardiovascular disease or its risk factors (32%, 47/149). Of these, studies specifically focused on hypertension (43%, 30/47), ischaemic heart disease (15%, 7/47), heart failure (13%, 6/47), and CVD risk factors in general (32%, 15/47). The second most common condition studied was mental health (32%, 48/149) which included depression, anxiety, and post-traumatic stress. eighteen percent of all studies focussed on diabetes (27/149). Cancer was investigated in 13% (20/149) of the included studies. Of these, interventions specifically focused on breast cancer (55%, 11/20), cervical cancer (30%, 6/20), and gastrointestinal cancer (15%, 3/20). Respiratory conditions included asthma and chronic obstructive pulmonary diseases (7%,10/149). One study focussed on the management of chronic kidney disease (1/149). Seventeen studies (11%) included two or more conditions, and 13 of this included mental health along with another chronic illness.

### Was task-sharing effective in improving health outcomes?

Out of the 149 studies, 120 studies (81%) reported a significant primary outcome (or at least 1 significant primary outcome), while 19 studies reported neutral results, and one reported a negative result. Table 1 highlights the effect of each outcome. All the task-sharing studies involving the care for cancer morbidity reported at least one positive primary outcome. Twelve of the 15 task-sharing studies which used a digital health intervention reported at least one positive primary outcome, while three reported non-statistically significant outcomes[48,54,59]. Outcomes of all task-sharing interventions are shown on Table 2. In studies where the primary outcome was not achieved, results demonstrated that task-sharing for NCD prevention and control was acceptable, feasible, and resulted in better treatment uptake[53,60–62].

**Fig 3:**
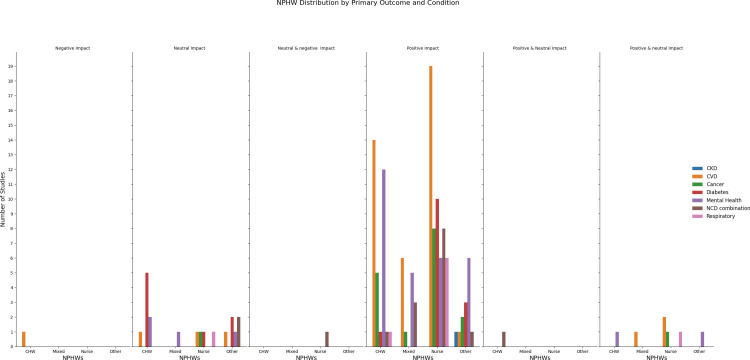
Number of studies by NCD conditions, types of NPHWs, and primary outcomes. Footnote. **Nurse**: Includes all roles involving nurses, midwives, and advanced practice nurses. **CHW**: Refers to community health workers, lay health workers, and similar roles. **Mixed**: Combines roles, such as CHWs and nurses, or interdisciplinary teams, including physicians. **Other**: Encompasses roles that do not fall into the above categories, such as dietitians and researchers.

Moreover, Fig 3 illustrates the distribution of studies across various NCD conditions, types of NPHWs, and their associated primary outcomes. A significant number of studies reported positive outcomes for CVD when CHWs and nurses were involved. Additionally, larger studies highlighted positive outcomes for mental health conditions with CHWs, while nurses showed positive results for diabetes management.

### What does process evaluation reveal about task-sharing?

The enablers of these studies included the context in which NPHWs carry out their activities, their relationship and trust with the community, support by the leadership and training provided[52,61,63,64]. Training was determined to be vital for these interventions as it provided NPHWs the necessary skills, knowledge, and confidence to deliver health care for NCDs[61, 62]. Another necessary enabler was the availability of resources such as equipment to measure blood pressure or strips to check blood glucose, and a regular medicine supply[63]. The use of digital health tools demonstrated quality improvement and provided standardised and evidence-based care to communities[53]. The simplicity of the intervention, leveraging existing infrastructure and resources, and a collaborative care model facilitated the intervention’s success[52,63]. Some studies provided free medicines or phones with prepaid data to support the implementation of the intervention. Nonetheless, the sustainability and scale-up of these interventions is debatable[52]. Support from senior managers and leaders was considered critical for the success of task-sharing interventions[63,65]. Legitimising the role of the NPHW to ensure community acceptability, especially as they provide new services for conditions such as NCDs was considered essential[61].

Key barriers included the non-availability and erratic supply of medicines in public health facilities [53,60], distrust in the medicines available [61], a lack of equipment [63] and long waiting times for the PHC[64]. Furthermore, interventions that required space for delivery such as counselling or patient education found that many PHCs prioritised conditions such as HIV over NCDs and did not allocate any space within the health facility[60,62,65]. Some evaluations identified challenges such as poor management processes, poor relationships between PHC workers and conflict with higher level occupational groups[60,63]. NPHWs navigated this barrier with the support of community leaders or influential stakeholders[62]. Transportation for patients to attend the PHC, and health workers to visit patients at home, especially those who did not reside in the same community was identified as another barrier[63, 64]. Nurses also felt the need to improve mechanisms to store patient information at the PHC[63].

### Is task-sharing a cost-effective intervention?

Thirteen studies reported the costs involved [31,32,40,52,66–68] or evaluated the cost-effectiveness [28,69–73] of the intervention. Most (7/13) of these studies were conducted for interventions relating to mental health [40,68,72,74], three studies for hypertension [70,71,74], two for diabetes [69,73] and one for cardiovascular disease[66]. Economic assessment for the management of depression demonstrated that the intervention was cost-effective, with the task-sharing intervention costing US$120 less in the intervention arm than in the control arm in public health facilities[40]. A multi-country study on psychosis reported larger reductions in overall healthcare costs in the intervention group than in the control group. The study reported higher cumulative costs over the intervention period (US $627 per patient vs $526 in the control group[31]. The incremental cost-effectiveness ratio for task-shared care in Ethiopia indicated lower cost of –US$299·82 (95% CI –454·95 to –144·69) per unit increase in severe mental disorder clinical symptom severity (calculated using a brief psychiatric rating scale) from the health sector perspective at 12 months[28]. However, one study showed that the treatment was more costly per participant per year (US$117.16, 95%CI 94.05, 140.26) compared to enhanced usual care (US$85.30, 95%CI 55.98, 114.62; p = 0.04) and not cost-effective[68].

Task-sharing interventions for diabetes and hypertension indicated that involved trained NPHWs were cost-effective[69,71,73]. The incremental cost-effectiveness ratio for the CHW intervention for diabetes in American Samoa was calculated at $13,191 per quality-adjusted life year (QALY) gained, which is considered highly cost-effective compared to commonly accepted willingness-to-pay thresholds ranging from $39,000 to $154,353 per QALY in the study context. Some of the studies also measured the costs of intervention[69]. A study in South Asia estimated the cost of scale-up of a CHW task-sharing intervention for hypertension to be US$10.70, US$10.50, and US$4.70 per individual in Bangladesh, Pakistan, and Sri Lanka respectively[32]. Another study which trained CHWs to provide management for CVD estimated costs per individual at high‐risk of CVD for three different models of care at 11 USD (CHW salary, training and physical measurement of CVD risk), 12 USD (basic model and medicines for CVD) and 14 USD (basic model, medicines and physician time)[66].

### Unintended consequences of task-sharing

As NPHWs were trained to take on new roles, in some contexts, this generated conflict with other staff[60,75]. For instance, studies in South Africa [60] and India [61] demonstrated that nurses were unhappy with the CHWs likely because their role was indirectly challenged by that of CHWs who were trained to perform tasks similar to theirs. Furthermore, some studies reported challenges relating to insufficient remuneration of the CHWs, especially as they took on new roles[75]. A study which evaluated the management of hypertension at the PHC level where services for chronic disease including HIV are provided, demonstrated that vertically funded programs such as HIV and the poor standards of equipment in clinics compromised the quality of services provided by nurses[76].

Training improved the confidence and communication style and skills of CHWs, though some CHWs offered unsolicited information to patients[65]. Using digital health tools and sharing tasks with the PHC doctor for a common goal to improve health outcomes-built legitimacy for the CHW’s new role[61].

### Risk of Bias of individual studies

Overall, over half (57%, 85/149) of studies had a low risk of bias, 8% (12/149) had a high risk of bias, and 35% (52/149) had some concerns with bias. There was a low risk of bias associated with randomisation (115/149), deviations (120/149), missing outcome data (129/149), measurement of outcomes (133/149), and selection of report result (131/149) in most studies. For full reporting of ROB results, please refer to S1 Appendix.

## Discussion

Our systematic review included studies that utilised NPHWs to prevent or control NCDs, and explored the barriers, facilitators and unexpected consequences of task-sharing. Our search identified 149 RCTs across 31 countries, of which 81% reported a positive primary outcome, demonstrating that task-sharing is an effective intervention for NCDs. NPHWs included CHWs, nurses, dietitians, nutritionists, and traditional faith healers. A sub-set of these studies which included economic analyses found that task-sharing can reduce the total costs of healthcare of patients with depression, anxiety, hypertension and diabetes and improve health outcomes in public facilities[28,69–72]. One study showed that task-sharing interventions were more costly than usual care [31], owing to the training and equipment required to upskill the workforce for providing quality health services.

Previous reviews on task-sharing have identified that it is effective for screening, prevention, and in some cases, the management of mental health conditions [77], hypertension [78], CVD [79], diabetes [80], cholesterol [81], cervical cancer [82] and other NCDs [11,83] - though effectiveness was not demonstrated for cholesterol-lowering interventions[81]. Task sharing has been achieved either by organising the available health workforce by expanding their current roles to include management of NCDs [53,54,74] or by employing additional resources such as community volunteers [84] or faith healers[31]. These models of care usually employ a multidisciplinary team of CHWs and nurses with or without physicians. Although our review found that 81% of the studies reported positive primary outcomes, indicating that task-sharing is an effective intervention for NCDs, a few studies reported neutral, mixed, and negative (one study) results. Various contextual factors at different levels seem to have contributed to these mixed outcomes. At the health system level, factors such as health infrastructure, the capabilities of NPHWs in implementing interventions, and human resource interventions (e.g., supervision and training) may affect effectiveness. Additionally, patient-level factors, such as engagement with interventions and adherence to treatment, also play a role in shaping the outcomes of task-sharing [48,54,59].

Furthermore, many of the task-sharing interventions were multifaceted, some aided by digital health to provide clinical decision support to the workforce[49,51,54,59], and others by the use of phone calls for health education, follow-up and medication adherence [55,85,86] to improve health outcomes. Some studies focussed on a single disease or risk factor [38,87,88] while others evaluated task-sharing for a range of conditions[46,53,89–93]. Use of technology, training, and supervision of the health workforce were identified as facilitators.

However, the effectiveness of NPHWs in task-sharing interventions for NCDs can be influenced by whether they are dedicated solely to a given intervention or tasked with multiple duties within the broader health system. Studies where NPHWs received focused training and were assigned well-defined roles generally report positive outcomes, as these workers can concentrate on NCD-related tasks without competing responsibilities [54]. While digital health-related interventions improved access to effective health care and improved patient outcomes in most studies, scaling up the intervention would require considerable planning and funding to avoid ‘pilotitis’[94]. Appreciation of the context and system-related issues such as non-availability of medicines or the need for a doctor to initiate treatment, as well as data integration with the sub-national or national health information system are important considerations.

As acknowledged by other reviews[11,83], a key takeaway was the macro-level and systemic barriers such as poor medicine supply, lack of equipment and infrastructure which impeded task-sharing. These issues directly influenced intervention outcomes, with some studies reporting disruptions in medication or equipment that hindered NPHWs’ ability to deliver care [11,83]. Such barriers highlight the need to address systemic challenges, including supply chain inefficiencies. Ensuring a consistent supply of essential drugs, establishing an efficient distribution system, and providing training on proper use are critical steps to enhance the effectiveness of task-sharing interventions, particularly in resource-constrained settings [11].

Additional factors included lack of trust in the ‘free medicines’ provided by the public health system[61], low priority given to NCDs compared to communicable diseases [60,62] and additional costs involved in home visits for follow-up[63]. In fact, a recent assessment of Ethiopia’s Health Extension Program services showed that better HIV program performance by CHWs was associated with lower uptake of NCD preventive services[95]. This finding supports the opinion that integration of new programs to existing service packages may spread resources too thinly[96]. This may jeopardise the success of existing health services resulting in worse health outcomes[95].

Having strong community engagement was found to circumvent some of these barriers[62]. As these multifaceted, complex, task-sharing interventions intrinsically depend on the interpersonal relationships of the healthcare teams, some studies found that if the roles of various team members were not clearly defined, it led to role conflict[60,75]. Other researchers have reported that the non-availability of protocols, lack of job description, differential financial incentives and the display of occupational superiority leads to role conflicts among the non-physician PHC team members[97].

Task-sharing is a well-accepted and an effective model of care which can help address the challenges of workforce shortages and inequities in healthcare access. The model has been embedded in the health system of several LMICs for decades to deliver care for communicable diseases and maternal and child health[98]. However, as communicable diseases require short-term care, adapting this model to address the long-term care needs of individuals with NCDs is essential. Decentralising services through task-sharing enables NPHWs to provide vital care in community settings, improving accessibility and continuity of care—key factors for managing chronic conditions effectively.

### Recommendations from this review

S3 Appendix documents detailed evidence about the tasks shared by each category of NPHWs for each type of NCD across the continuum of care (prevention, diagnosis, management, and rehabilitation) of patients with NCDs. Using a systems lens with a focus on task-sharing for NCDs in LMICs, we have the following recommendations. At the **macro level**, national health policies need to include a specific policy for NCD related prevention, promotion, management, rehabilitation, and palliation. In order to implement these policies, countries need to invest in NCDs and allocate sufficient funds. As these NPHWs are also tasked with delivering additional services other than NCDs, including those related to communicable diseases, there is a need for health systems to focus on effective integration of services and systems.

At the **meso level**, implementors should ideally move from small scale pilots and trials to scaling up evidence-based solutions such as WHO Best buys through PHC as the platform for NCD care. Evidence demonstrates that digital health tools assist the health workforce to provide quality and standardised care and legitimises the role of CHWs. Availability of equipment, regular medicine supply and adequate space are necessary to build workforce and community trust in the health system. Furthermore, to motivate and retain the workforce, they need to be adequately remunerated.

At the **micro level**, our review highlights that all occupational groups need to have clear job descriptions with appropriate training and retraining of health workforce, especially NPHWs (e.g. CHWs, nurses) to improve their confidence, knowledge and skill set for basic NCD management at the community level. Accountability and community engagement were found to facilitate services. As team-based care requires close interaction and trust between team members, it is essential to provide guidance about how services will be integrated and how each occupation will function. The capability, opportunity, and motivation (COM-B) theory uses three interrelated domains which are linked to behaviour change. Capability includes physical and psychological capacity to engage in or perform an activity, motivation refers to automated and reflective brain processes that energize and direct behaviour, and opportunity refers to all the factors that lie outside of the control of an individual that influence change [75].

### Strengths and limitations

Our review includes all randomised controlled trials on task-sharing for NCD management reported in peer reviewed English, Spanish and French language journals. The strength of our report lies in its comprehensive scope as it is a large review encompassing a range of NPHWs and publications reporting both positive and neutral primary outcomes. It explored the barriers, facilitators, and unexpected consequences of task-sharing to NPHWs in the prevention and control of NCDs. Additionally, the overall risk of bias was low in majority of the studies included.

However, the study is not without limitations, most of which stem from the broader scope of the review, which may have missed specific details and nuances. One is the exclusion of studies published in languages other than English, Spanish, and French, which may have led to missing relevant examples from larger nations where research is conducted in other languages. Another limitation is related to combining all countries under the broad label of “LMICs,” which overlooks critical differences in health systems, economic conditions, and cultural and social environments. A more detailed analysis of how task-sharing varies across specific regions and contexts would further enrich the findings. Additionally, very few RCTs reported process evaluation data, which is essential to understand the contextual factors and fidelity of the intervention. Details about training or retraining of the workforce, their supervision and remuneration was not discussed in the studies. Moreover, the diverse reporting in the included studies meant that we could not report disaggregated outcomes contributed by specific NPHWs. The other limitation of this review is that all the findings are based within a research context, which is usually challenging to scale-up due to a number of reasons including cost, adequate monitoring, health inequities, challenges in implementation due to shortage of workforce[99]. Additionally, the exclusion of implementation research may have led to the omission of important contextual and real-world evidence that could provide valuable insights into the practical application of task-sharing interventions. Furthermore, few studies reported cost-effectiveness data which is important for assessing budget impact and the feasibility of scaling sustainably. Nonetheless, the study successfully attained its objectives as it focused on the task-sharing practices of NPHWs with different levels of training [9].

## Conclusions

Our review demonstrates that using task-sharing models of care involving trained NPHWs is effective and cost-effective in LMICs where NCDs are the leading causes of premature deaths and disability. Ultimately, task-sharing, should not be viewed as a task-dumping exercise to the ‘lowest’ occupational group, on the contrary, it ought to be designed and implemented as a team-based approach where all members are motivated, trained, remunerated and have the government and community’s support to deliver their roles to the best of their ability.

## Supporting information

S1 Appendix – Search Terms.
(PDF)

S2 Appendix – Risk of bias results.
(PDF)

S3 Appendix – Evidence of effective task-sharing from this review.
(PDF)
