# Supplementary material for: Task-sharing for non-communicable disease prevention and control in low- and middle-income countries in the context of health worker shortages: A systematic review
Source: PLOS Glob Public Health. 2025 Apr 16;5(4):e0004289. doi: 10.1371/journal.pgph.0004289 (PMC12002516; doi:10.1371/journal.pgph.0004289)
Supplement: S1 Appendix – — Search Terms. (PDF) [file pgph.0004289.s001.pdf]

S1 Appendix – Search Terms

Pubmed

(("health personnel"[MeSH Terms] OR "health auxiliary"[All Fields] OR "chw"[All Fields] OR "community health workers"[MeSH Terms] OR "physician assistants"[MeSH Terms] OR ("nurse administrators"[MeSH Terms] OR "nurse clinicians"[MeSH Terms] OR "nurse midwives"[MeSH Terms] OR "nurse specialists"[MeSH Terms] OR "nurse practitioners"[MeSH Terms]) OR "health extension worker"[All Fields] OR ("community health extension worker"[All Fields] OR "community health extension workers"[All Fields]) OR ("auxiliary nurse midw*"[All Fields] OR "auxiliary nurses"[All Fields] OR "auxiliary nurs*"[All Fields] OR "auxiliary nurses and midwives"[All Fields]) OR "medical assistant"[All Fields] OR "clinical associate"[All Fields] OR ("task shar*"[All Fields] OR "task shift*"[All Fields])) AND

((("cardiovascular diseases"[MeSH Terms] OR "cardiovascular risk"[All Fields] OR "cvd"[All Fields] OR ("cancer"[All Fields] OR "neoplasm"[All Fields] OR "malignan*"[All Fields] OR "malignancy"[All Fields]) OR ("respiratory disease*"[All Fields] OR "respiratory tract diseases"[MeSH Terms]) OR ((("asthma"[MeSH Terms] OR "asthma chronic obstructive pulmonary disease overlap syndrome"[MeSH Terms] OR "bronchial asthma"[All Fields] OR "severe asthma"[All Fields] OR "exercise induced asthma"[All Fields] OR "extrinsic asthma"[All Fields] OR "allergic asthma"[All Fields] OR "nocturnal asthma"[All Fields] OR "moderate persistent asthma"[All Fields] OR "mild persistent asthma"[All Fields] OR "cardiac asthma"[All Fields]) AND ("randomized controlled trial"[Publication Type] AND "humans"[MeSH Terms] AND ("english"[Language] OR "french"[Language] OR "spanish"[Language]))) OR ("copd"[All Fields] OR "chronic obstructive lung disease"[All Fields] OR "chronic obstructive airway disease"[All Fields]) OR ("mental health"[MeSH Terms] OR "mental health"[All Fields]) OR ("depression"[All Fields] OR "depression"[MeSH Terms] OR ("mixed manic depressive"[All Fields] OR "mixed manic depressive states"[All Fields]) OR "recurrent brief depression"[All Fields] OR "inbreeding depression"[All Fields] OR "treatment resistant depression"[All Fields] OR "postoperative depression"[All Fields] OR "bipolar depression"[All Fields] OR "masked depression"[All Fields] OR "minor depression"[All Fields] OR "involutional depression"[All Fields] OR "depression assessment"[All Fields] OR "atypical depression"[All Fields] OR "adolescent depression"[All Fields] OR "long term depression"[All Fields] OR "postnatal depression"[All Fields] OR "endogenous depression"[All Fields] OR "mixed depression anxiety"[All Fields] OR "late life depression"[All Fields] OR "antenatal depression"[All Fields] OR "organic depression"[All Fields] OR "perinatal depression"[All Fields] OR "reactive depression"[All Fields] OR "chronic depression"[All Fields] OR "agitated depression"[All Fields] OR "major depression"[All Fields] OR "mild depression"[All Fields]) OR ("anxiety"[MeSH Terms] OR "anxiety disorders"[MeSH Terms] OR "generalised anxiety disorder"[All Fields] OR ("anxiety"[All Fields] OR "anxiety and depression"[All Fields]) OR "generalised anxiety disorder"[All Fields] OR "anxiety assessment"[All Fields] OR "anxiety neurosis"[All Fields]))) AND

("afghan"[Text Word] OR "afghans"[Text Word] OR "afghani"[Text Word] OR "albanian"[Text Word] OR "albanians"[Text Word] OR "algerian"[Text Word] OR "algerians"[Text Word] OR "american samoan"[Text Word] OR "american samoans"[Text Word] OR "angolan"[Text Word] OR "angolans"[Text Word] OR "antiguan"[Text Word] OR "antiguans"[Text Word] OR "barbudan"[Text Word] OR "argentine"[Text Word] OR "argentines"[Text Word] OR "argentinian"[Text Word] OR "argentinians"[Text Word] OR "argentinean"[Text Word] OR "argentineans"[Text Word] OR "armenian"[Text Word] OR "armenians"[Text Word] OR "aruban"[Text Word] OR "arubans"[Text Word] OR "azerbaijani"[Text Word] OR "azerbaijanis"[Text Word] OR "bahraini"[Text Word] OR "bahrainis"[Text Word] OR "bangladeshi"[Text Word] OR "bangladeshis"[Text Word] OR "bangalees"[Text Word] OR "bajan"[Text Word] OR "belarusian"[Text Word] OR "belarusians"[Text Word] OR "byelorussian"[Text Word] OR "byelorussians"[Text Word] OR "belizean"[Text Word] OR "belizeans"[Text Word] OR "beninese"[Text Word] OR "benineses"[Text Word] OR "bhutanese"[Text Word] OR "bolivian"[Text Word] OR "bolivians"[Text Word] OR "bosnian"[Text Word] OR "bosnians"[Text Word] OR "botswana"[Text Word] OR "batswana"[Text Word] OR "brazilian"[Text Word] OR "brazilians"[Text Word] OR "brasilian"[Text Word] OR "brasilians"[Text Word] OR "bulgarian"[Text Word] OR "bulgarians"[Text Word] OR "burkinabe"[Text Word] OR "burundian"[Text Word] OR "burundians"[Text Word] OR "cape verdean"[Text Word] OR "cape verdeans"[Text Word] OR (("cabo"[Journal] OR "cabo"[All Fields]) AND "verdean"[Text Word]) OR "cabo verdeans"[Text Word] OR "cambodian"[Text Word] OR "cambodians"[Text Word] OR "khmer"[Text Word] OR "cameroonian"[Text Word] OR "cameroonians"[Text Word] OR "central african"[Text Word] OR "central africans"[Text Word] OR "chadian"[Text Word] OR "chadians"[Text Word] OR "chilean"[Text Word] OR "chileans"[Text Word] OR "chinese"[Text Word] OR "colombian"[Text Word] OR "colombians"[Text Word] OR "comorian"[Text Word] OR "comorians"[Text Word] OR "congolese"[Text Word] OR "costa rican"[Text Word] OR "costa ricans"[Text Word] OR "ivorian"[Text Word] OR "ivorians"[Text Word] OR "croatian"[Text Word] OR "croatians"[Text Word] OR "cuban"[Text Word] OR "cubans"[Text Word] OR "cypriot"[Text Word] OR "cypriots"[Text Word] OR "czech"[Text Word] OR "czechs"[Text Word] OR "djiboutian"[Text Word] OR "djiboutians"[Text Word] OR "dominican"[Text Word] OR "dominicans"[Text Word] OR "ecuadorian"[Text Word] OR "ecuadorians"[Text Word] OR "egyptian"[Text Word] OR "egyptians"[Text Word] OR "salvadoran"[Text Word] OR "salvadorans"[Text Word] OR "equatorial guinean"[Text Word] OR (("equatorial"[All Fields] OR "equatorially"[All Fields] OR "equatorials"[All Fields]) AND "guineans"[Text Word]) OR "equatoguinean"[Text Word] OR "equatoguineans"[Text Word] OR "eritrean"[Text Word] OR "eritreans"[Text Word] OR "estonian"[Text Word] OR "estonians"[Text Word] OR "swazi"[Text Word] OR "swazis"[Text Word] OR "swati"[Text Word] OR "ethiopian"[Text Word] OR "ethiopians"[Text Word] OR "fijian"[Text Word] OR "fijians"[Text Word] OR "gabonese"[Text Word] OR "gambian"[Text Word] OR "gambians"[Text Word] OR "georgian"[Text Word] OR "georgians"[Text Word] OR "ghanaian"[Text Word] OR "ghanaians"[Text Word] OR "gibraltarian"[Text Word] OR

"gibraltarians"[Text Word] OR "greek"[Text Word] OR "greeks"[Text Word] OR "grenadian"[Text Word] OR "grenadians"[Text Word] OR "guamanian"[Text Word] OR "guamanians"[Text Word] OR "guatemalan"[Text Word] OR "guatemalans"[Text Word] OR "guinean"[Text Word] OR "guineans"[Text Word] OR "bissau guinean"[Text Word] OR "bissau guineans"[Text Word] OR "guyanese"[Text Word] OR "haitian"[Text Word] OR "haitians"[Text Word] OR "honduran"[Text Word] OR "hondurans"[Text Word] OR "hungarian"[Text Word] OR "hungarians"[Text Word] OR "indian"[Text Word] OR "indians"[Text Word] OR "indonesian"[Text Word] OR "indonesians"[Text Word] OR "iranian"[Text Word] OR "iranians"[Text Word] OR "iraqian"[Text Word] OR "iraqi"[Text Word] OR "iraqis"[Text Word] OR "manx"[Text Word] OR "jamaican"[Text Word] OR "jamaicans"[Text Word] OR "jordanian"[Text Word] OR "jordanians"[Text Word] OR "kazakhstani"[Text Word] OR "kazakhstanis"[Text Word] OR "kenyan"[Text Word] OR "kenyans"[Text Word] OR "korean"[Text Word] OR "koreans"[Text Word] OR "kosovar"[Text Word] OR "kosovars"[Text Word] OR "kosovan"[Text Word] OR "kosovans"[Text Word] OR "kyrgyzstani"[Text Word] OR "kyrgyz"[Text Word] OR "lao"[Text Word] OR "laotian"[Text Word] OR "laotians"[Text Word] OR "latvian"[Text Word] OR "latvians"[Text Word] OR "lebanese"[Text Word] OR "mosotho"[Text Word] OR "basotho"[Text Word] OR "liberian"[Text Word] OR "liberians"[Text Word] OR "libyan"[Text Word] OR "libyans"[Text Word] OR "lithuanian"[Text Word] OR "lithuanians"[Text Word] OR "macanese"[Text Word] OR "macedonian"[Text Word] OR "macedonians"[Text Word] OR "malagasy"[Text Word] OR "madagascan"[Text Word] OR "madagascans"[Text Word] OR "malawian"[Text Word] OR "malawians"[Text Word] OR "malaysian"[Text Word] OR "malaysians"[Text Word] OR "maldivian"[Text Word] OR "maldivians"[Text Word] OR "malian"[Text Word] OR "malians"[Text Word] OR "maltese"[Text Word] OR "marshallese"[Text Word] OR "mauritanian"[Text Word] OR "mauritanians"[Text Word] OR "mauritian"[Text Word] OR "mauritians"[Text Word] OR "mexican"[Text Word] OR "mexicans"[Text Word] OR "micronesian"[Text Word] OR "micronesians"[Text Word] OR "moldovan"[Text Word] OR "moldovans"[Text Word] OR "mongolian"[Text Word] OR "mongolians"[Text Word] OR "mongol"[Text Word] OR "montenegrin"[Text Word] OR "montenegrins"[Text Word] OR "moroccan"[Text Word] OR "moroccans"[Text Word] OR "mozambican"[Text Word] OR "mozambicans"[Text Word] OR "burmese"[Text Word] OR "myanma"[Text Word] OR "namibian"[Text Word] OR "namibians"[Text Word] OR "nauruan"[Text Word] OR "nauruans"[Text Word] OR "nepali"[Text Word] OR "nepalese"[Text Word] OR "netherlands antillean"[Text Word] OR "netherlands antilleans"[Text Word] OR "nicaraguan"[Text Word] OR "nicaraguans"[Text Word] OR "nigerien"[Text Word] OR "nigeriens"[Text Word] OR "nigerian"[Text Word] OR "nigerians"[Text Word] OR ((("northern"[All Fields] OR "northerners"[All Fields] OR "northerns"[All Fields]) AND ("mariana"[All Fields] OR "marianas"[All Fields])) AND "islander"[Text Word]) OR (("northern"[All Fields] OR "northerners"[All Fields] OR "northerns"[All Fields]) AND "mariana islanders"[Text Word]) OR "mariana"[Text Word] OR "marianas"[Text Word] OR "omani"[Text Word] OR "omanis"[Text Word] OR "pakistani"[Text Word] OR "pakistanis"[Text Word] OR

"palauan"[Text Word] OR "palauans"[Text Word] OR "panamanian"[Text Word] OR "panamanians"[Text Word] OR "papua new guinean"[Text Word] OR "papua new guineans"[Text Word] OR "paraguayan"[Text Word] OR "paraguayans"[Text Word] OR "peruvian"[Text Word] OR "peruvians"[Text Word] OR "philippine"[Text Word] OR "philippines"[Text Word] OR "philipine"[Text Word] OR "philipines"[Text Word] OR "phillipine"[Text Word] OR "phillipines"[Text Word] OR "phillippine"[Text Word] OR "phillippines"[Text Word] OR "filipino"[Text Word] OR "filipinos"[Text Word] OR "filipina"[Text Word] OR "filipinas"[Text Word] OR "polish"[Text Word] OR "pole"[Text Word] OR "poles"[Text Word] OR "portuguese"[Text Word] OR "puerto rican"[Text Word] OR "puerto ricans"[Text Word] OR "romanian"[Text Word] OR "romanians"[Text Word] OR "russian"[Text Word] OR "russians"[Text Word] OR "soviet people"[Text Word] OR "soviet population"[Text Word] OR "rwandan"[Text Word] OR "rwandans"[Text Word] OR "rwandese"[Text Word] OR "ruandans"[Text Word] OR "ruandese"[Text Word] OR "samoan"[Text Word] OR "samoans"[Text Word] OR ("sao"[All Fields] AND "tomean"[Text Word]) OR "sao tomeans"[Text Word] OR "saudi arabian"[Text Word] OR "saudi arabians"[Text Word] OR "saudi"[Text Word] OR "saudis"[Text Word] OR "senegalese"[Text Word] OR "serbian"[Text Word] OR "serbians"[Text Word] OR "montenegrin"[Text Word] OR "montenegrins"[Text Word] OR "seychellois"[Text Word] OR "seychelloise"[Text Word] OR "sierra leonean"[Text Word] OR "sierra leoneans"[Text Word] OR "slovak"[Text Word] OR "slovaks"[Text Word] OR "slovene"[Text Word] OR "slovenes"[Text Word] OR "solomon islander"[Text Word] OR "solomon islanders"[Text Word] OR "somali"[Text Word] OR "somalis"[Text Word] OR "south african"[Text Word] OR "south africans"[Text Word] OR "south sudanese"[Text Word] OR "sri lankan"[Text Word] OR "sri lankans"[Text Word] OR "ceylonese"[Text Word] OR "kittitian"[Text Word] OR "kittitians"[Text Word] OR "nevisian"[Text Word] OR "saint lucian"[Text Word] OR (("saint s"[All Fields] OR "sainte"[All Fields] OR "saints"[MeSH Terms] OR "saints"[All Fields] OR "saint"[All Fields]) AND "lucians"[Text Word]) OR "vincentian"[Text Word] OR "vincentians"[Text Word] OR "sudanese"[Text Word] OR "surinamese"[Text Word] OR "syrian"[Text Word] OR "syrians"[Text Word] OR "tajik"[Text Word] OR "tajiks"[Text Word] OR "tajikistani"[Text Word] OR "tanzanian"[Text Word] OR "tanzanians"[Text Word] OR "tanganyikan"[Text Word] OR "thai"[Text Word] OR "timorese"[Text Word] OR "timoreses"[Text Word] OR "togolese"[Text Word] OR "tongan"[Text Word] OR "tongans"[Text Word] OR "trinidadian"[Text Word] OR "trinidadians"[Text Word] OR "tobagonian"[Text Word] OR "tobagonians"[Text Word] OR "tunisian"[Text Word] OR "tunisians"[Text Word] OR "turk"[Text Word] OR "turks"[Text Word] OR "turkish"[Text Word] OR "turkmen"[Text Word] OR "turkmens"[Text Word] OR "tuvaluan"[Text Word] OR "tuvaluans"[Text Word] OR "ugandan"[Text Word] OR "ugandans"[Text Word] OR "ukrainian"[Text Word] OR "ukrainians"[Text Word] OR "uruguayan"[Text Word] OR "uruguayans"[Text Word] OR "uzbek"[Text Word] OR "uzbeks"[Text Word] OR "vanuatu"[Text Word] OR "vanuatuan"[Text Word] OR "vanuatuans"[Text Word] OR "venezuelan"[Text Word] OR "venezuelans"[Text Word] OR "vietnamese"[Text Word] OR "yemeni"[Text Word] OR

"yemenis"[Text Word] OR "yemenite"[Text Word] OR "yemenites"[Text Word] OR "yemenese"[Text Word] OR "yugoslav"[Text Word] OR "yugoslavs"[Text Word] OR "yugoslavian"[Text Word] OR "yugoslavians"[Text Word] OR "zambian"[Text Word] OR "zambians"[Text Word] OR "zimbabwean"[Text Word] OR "zimbabweans"[Text Word] OR ((("afghanistan"[MeSH Terms] OR "albania"[MeSH Terms] OR "algeria"[MeSH Terms] OR "american samoa"[MeSH Terms] OR "angola"[MeSH Terms] OR "antigua and barbuda"[MeSH Terms] OR "argentina"[MeSH Terms] OR "armenia"[MeSH Terms] OR "aruba"[MeSH Terms] OR "azerbaijan"[MeSH Terms] OR "bahrain"[MeSH Terms] OR "bangladesh"[MeSH Terms] OR "barbados"[MeSH Terms] OR "republic of belarus"[MeSH Terms] OR "belize"[MeSH Terms] OR "benin"[MeSH Terms] OR "bhutan"[MeSH Terms] OR "bolivia"[MeSH Terms] OR "bosnia and herzegovina"[MeSH Terms] OR "botswana"[MeSH Terms] OR "brazil"[MeSH Terms] OR "bulgaria"[MeSH Terms] OR "burkina faso"[MeSH Terms] OR "burundi"[MeSH Terms] OR "cabo verde"[MeSH Terms] OR "cambodia"[MeSH Terms] OR "cameroon"[MeSH Terms] OR "central african republic"[MeSH Terms] OR "chad"[MeSH Terms] OR "chile"[MeSH Terms] OR "china"[MeSH Terms] OR "colombia"[MeSH Terms] OR "comoros"[MeSH Terms] OR "democratic republic of the congo"[MeSH Terms] OR "congo"[MeSH Terms] OR "costa rica"[MeSH Terms] OR "cote d ivoire"[MeSH Terms] OR "croatia"[MeSH Terms] OR "cuba"[MeSH Terms] OR "cyprus"[MeSH Terms] OR "czech republic"[MeSH Terms] OR "djibouti"[MeSH Terms] OR "dominica"[MeSH Terms] OR "dominican republic"[MeSH Terms] OR "ecuador"[MeSH Terms] OR "egypt"[MeSH Terms] OR "el salvador"[MeSH Terms] OR "equatorial guinea"[MeSH Terms] OR "eritrea"[MeSH Terms] OR "estonia"[MeSH Terms] OR "eswatini"[MeSH Terms] OR "ethiopia"[MeSH Terms] OR "fiji"[MeSH Terms] OR "gabon"[MeSH Terms] OR "gambia"[MeSH Terms] OR ("georgia"[MeSH Terms] OR "georgia"[All Fields] OR "georgia republic"[MeSH Terms] OR ("georgia"[All Fields] AND "republic"[All Fields]) OR "georgia republic"[All Fields] OR "georgia s"[All Fields])) AND ("republic"[All Fields] OR "republic s"[All Fields] OR "republics"[All Fields])) OR "ghana"[MeSH Terms] OR "gibraltar"[MeSH Terms] OR "greece"[MeSH Terms] OR "grenada"[MeSH Terms] OR "guam"[MeSH Terms] OR "guatemala"[MeSH Terms] OR "guinea"[MeSH Terms] OR "guinea bissau"[MeSH Terms] OR "guyana"[MeSH Terms] OR "haiti"[MeSH Terms] OR "honduras"[MeSH Terms] OR "hungary"[MeSH Terms] OR "india"[MeSH Terms] OR "indonesia"[MeSH Terms] OR "iran"[MeSH Terms] OR "iraq"[MeSH Terms] OR "jamaica"[MeSH Terms] OR "jordan"[MeSH Terms] OR "kazakhstan"[MeSH Terms] OR "kenya"[MeSH Terms] OR "democratic people s republic of korea"[MeSH Terms] OR "republic of korea"[MeSH Terms] OR "kosovo"[MeSH Terms] OR "kyrgyzstan"[MeSH Terms] OR "laos"[MeSH Terms] OR "latvia"[MeSH Terms] OR "lebanon"[MeSH Terms] OR "lesotho"[MeSH Terms] OR "liberia"[MeSH Terms] OR "libya"[MeSH Terms] OR "lithuania"[MeSH Terms] OR "macau"[MeSH Terms] OR "republic of north macedonia"[MeSH Terms] OR "madagascar"[MeSH Terms] OR "malawi"[MeSH Terms] OR "malaysia"[MeSH Terms] OR "indian ocean islands"[MeSH Terms] OR "mali"[MeSH Terms] OR "malta"[MeSH Terms] OR "micronesia"[MeSH Terms] OR "palau"[MeSH Terms] OR

"mauritania"[MeSH Terms] OR "mauritius"[MeSH Terms] OR "mexico"[MeSH Terms] OR "moldova"[MeSH Terms] OR "mongolia"[MeSH Terms] OR "montenegro"[MeSH Terms] OR "morocco"[MeSH Terms] OR "mozambique"[MeSH Terms] OR "myanmar"[MeSH Terms] OR "namibia"[MeSH Terms] OR "nepal"[MeSH Terms] OR "netherlands antilles"[MeSH Terms] OR "nicaragua"[MeSH Terms] OR "niger"[MeSH Terms] OR "nigeria"[MeSH Terms] OR "oman"[MeSH Terms] OR "pakistan"[MeSH Terms] OR "panama"[MeSH Terms] OR "papua new guinea"[MeSH Terms] OR "paraguay"[MeSH Terms] OR "peru"[MeSH Terms] OR "philippines"[MeSH Terms] OR "poland"[MeSH Terms] OR "portugal"[MeSH Terms] OR "puerto rico"[MeSH Terms] OR "romania"[MeSH Terms] OR "russia"[MeSH Terms] OR "rwanda"[MeSH Terms] OR "samoa"[MeSH Terms] OR "sao tome and principe"[MeSH Terms] OR "saudi arabia"[MeSH Terms] OR "senegal"[MeSH Terms] OR "serbia"[MeSH Terms] OR "seychelles"[MeSH Terms] OR "sierra leone"[MeSH Terms] OR "slovakia"[MeSH Terms] OR "slovenia"[MeSH Terms] OR "melanesia"[MeSH Terms] OR "somalia"[MeSH Terms] OR "south africa"[MeSH Terms] OR "south sudan"[MeSH Terms] OR "sri lanka"[MeSH Terms] OR "saint kitts and nevis"[MeSH Terms] OR "saint lucia"[MeSH Terms] OR "saint vincent and the grenadines"[MeSH Terms] OR "sudan"[MeSH Terms] OR "suriname"[MeSH Terms] OR "syria"[MeSH Terms] OR "tajikistan"[MeSH Terms] OR "tanzania"[MeSH Terms] OR "thailand"[MeSH Terms] OR "timor leste"[MeSH Terms] OR "togo"[MeSH Terms] OR "tonga"[MeSH Terms] OR "trinidad and tobago"[MeSH Terms] OR "tunisia"[MeSH Terms] OR "turkey"[MeSH Terms] OR "turkmenistan"[MeSH Terms] OR "uganda"[MeSH Terms] OR "ukraine"[MeSH Terms] OR "uruguay"[MeSH Terms] OR "uzbekistan"[MeSH Terms] OR "vanuatu"[MeSH Terms] OR "venezuela"[MeSH Terms] OR "vietnam"[MeSH Terms] OR "middle east"[MeSH Terms] OR "yemen"[MeSH Terms] OR "yugoslavia"[MeSH Terms] OR "zambia"[MeSH Terms] OR "zimbabwe"[MeSH Terms] OR "africa south of the sahara"[MeSH Terms] OR "africa, central"[MeSH Terms] OR "africa, northern"[MeSH Terms] OR "africa, southern"[MeSH Terms] OR "africa, eastern"[MeSH Terms] OR "africa, western"[MeSH Terms] OR "west indies"[MeSH Terms] OR "indian ocean islands"[MeSH Terms] OR "caribbean region"[MeSH Terms] OR "central america"[MeSH Terms] OR "latin america"[MeSH Terms] OR "south america"[MeSH Terms] OR "asia, central"[MeSH Terms] OR "asia, northern"[MeSH Terms] OR "asia, southeastern"[MeSH Terms] OR "asia, western"[MeSH Terms] OR "europe, eastern"[MeSH Terms] OR "developing countries"[MeSH Terms]))) OR ("afghanistan"[Text Word] OR "albania"[Text Word] OR "algeria"[Text Word] OR "american samoa"[Text Word] OR "angola"[Text Word] OR "antigua"[Text Word] OR "barbuda"[Text Word] OR "argentina"[Text Word] OR "armenia"[Text Word] OR "armenian"[Text Word] OR "aruba"[Text Word] OR "azerbaijan"[Text Word] OR "bahrain"[Text Word] OR "bangladesh"[Text Word] OR "barbados"[Text Word] OR "belarus"[Text Word] OR "byelarus"[Text Word] OR "belorussia"[Text Word] OR "byelorussian"[Text Word] OR "belize"[Text Word] OR "british honduras"[Text Word] OR "benin"[Text Word] OR "dahomey"[Text Word] OR "bhutan"[Text Word] OR "bolivia"[Text Word] OR "bosnia"[Text Word] OR "herzegovina"[Text Word] OR "botswana"[Text Word] OR "bechuanaland"[Text Word] OR "brazil"[Text Word] OR

"brasil"[Text Word] OR "bulgaria"[Text Word] OR "burkina faso"[Text Word] OR "burkina fasso"[Text Word] OR "upper volta"[Text Word] OR "burundi"[Text Word] OR "urundi"[Text Word] OR "cabo verde"[Text Word] OR "cape verde"[Text Word] OR "cambodia"[Text Word] OR "kampuchea"[Text Word] OR "khmer republic"[Text Word] OR "cameroon"[Text Word] OR "cameron"[Text Word] OR "cameroun"[Text Word] OR "central african republic"[Text Word] OR "ubangi shari"[Text Word] OR "chad"[Text Word] OR "chile"[Text Word] OR "china"[Text Word] OR "colombia"[Text Word] OR "comoros"[Text Word] OR "comoro islands"[Text Word] OR "mayotte"[Text Word] OR "congo"[Text Word] OR "zaire"[Text Word] OR "costa rica"[Text Word] OR "cote d ivoire"[Text Word] OR "cote d ivoire"[Text Word] OR ("cote"[All Fields] AND "divoire"[Text Word]) OR "cote d ivoire"[Text Word] OR "ivory coast"[Text Word] OR "croatia"[Text Word] OR "cuba"[Text Word] OR "cyprus"[Text Word] OR "czech republic"[Text Word] OR "czechoslovakia"[Text Word] OR "djibouti"[Text Word] OR "french somaliland"[Text Word] OR "dominica"[Text Word] OR "dominican republic"[Text Word] OR "ecuador"[Text Word] OR "egypt"[Text Word] OR "united arab republic"[Text Word] OR "el salvador"[Text Word] OR "equatorial guinea"[Text Word] OR "spanish guinea"[Text Word] OR "eritrea"[Text Word] OR "estonia"[Text Word] OR "eswatini"[Text Word] OR "swaziland"[Text Word] OR "ethiopia"[Text Word] OR "fiji"[Text Word] OR "gabon"[Text Word] OR "gabonese republic"[Text Word] OR "gambia"[Text Word] OR "georgia"[Text Word] OR "georgian"[Text Word] OR "ghana"[Text Word] OR "gold coast"[Text Word] OR "gibraltar"[Text Word] OR "greece"[Text Word] OR "grenada"[Text Word] OR "guam"[Text Word] OR "guatemala"[Text Word] OR "guinea"[Text Word] OR "guyana"[Text Word] OR "guiana"[Text Word] OR "haiti"[Text Word] OR "hispaniola"[Text Word] OR "honduras"[Text Word] OR "hungary"[Text Word] OR "india"[Text Word] OR "indonesia"[Text Word] OR "timor"[Text Word] OR "iran"[Text Word] OR "iraq"[Text Word] OR "isle of man"[Text Word] OR "jamaica"[Text Word] OR "jordan"[Text Word] OR "kazakhstan"[Text Word] OR "kazakh"[Text Word] OR "kenya"[Text Word] OR "korea"[Text Word] OR "kosovo"[Text Word] OR "kyrgyzstan"[Text Word] OR "kirghizia"[Text Word] OR "kirgizstan"[Text Word] OR "kyrgyz republic"[Text Word] OR "kirghiz"[Text Word] OR "laos"[Text Word] OR "lao pdr"[Text Word] OR "lao people s democratic republic"[Text Word] OR "latvia"[Text Word] OR "lebanon"[Text Word] OR "lesotho"[Text Word] OR "basutoland"[Text Word] OR "liberia"[Text Word] OR "libya"[Text Word] OR "libyan arab jamahiriya"[Text Word] OR "lithuania"[Text Word] OR "macau"[Text Word] OR "macao"[Text Word] OR "macedonia"[Text Word] OR "madagascar"[Text Word] OR "malagasy republic"[Text Word] OR "malawi"[Text Word] OR "nyasaland"[Text Word] OR "malaysia"[Text Word] OR "maldives"[Text Word] OR "indian ocean"[Text Word] OR "mali"[Text Word] OR "malta"[Text Word] OR "micronesia"[Text Word] OR "kiribati"[Text Word] OR "marshall islands"[Text Word] OR "nauru"[Text Word] OR "northern mariana islands"[Text Word] OR "palau"[Text Word] OR "tuvalu"[Text Word] OR "mauritania"[Text Word] OR "mauritius"[Text Word] OR "mexico"[Text Word] OR "moldova"[Text Word] OR "moldovian"[Text Word] OR "mongolia"[Text Word] OR "montenegro"[Text Word] OR "morocco"[Text Word] OR "ifni"[Text Word] OR "mozambique"[Text Word] OR "portuguese

east africa"[Text Word] OR "myanmar"[Text Word] OR "burma"[Text Word] OR "namibia"[Text Word] OR "nepal"[Text Word] OR "netherlands antilles"[Text Word] OR "nicaragua"[Text Word] OR "niger"[Text Word] OR "nigeria"[Text Word] OR "oman"[Text Word] OR "muscat"[Text Word] OR "pakistan"[Text Word] OR "panama"[Text Word] OR "papua new guinea"[Text Word] OR "paraguay"[Text Word] OR "peru"[Text Word] OR "philippines"[Text Word] OR "philipines"[Text Word] OR "phillipines"[Text Word] OR "phillippines"[Text Word] OR "poland"[Text Word] OR "polish people s republic"[Text Word] OR "portugal"[Text Word] OR "portuguese republic"[Text Word] OR "puerto rico"[Text Word] OR "romania"[Text Word] OR "russia"[Text Word] OR "russian federation"[Text Word] OR "ussr"[Text Word] OR "soviet union"[Text Word] OR "union of soviet socialist republics"[Text Word] OR "rwanda"[Text Word] OR "ruanda"[Text Word] OR "samoa"[Text Word] OR "pacific islands"[Text Word] OR "polynesia"[Text Word] OR "samoan islands"[Text Word] OR "sao tome and principe"[Text Word] OR "saudi arabia"[Text Word] OR "senegal"[Text Word] OR "serbia"[Text Word] OR "seychelles"[Text Word] OR "sierra leone"[Text Word] OR "slovakia"[Text Word] OR "slovak republic"[Text Word] OR "slovenia"[Text Word] OR "melanesia"[Text Word] OR "solomon island"[Text Word] OR "solomon islands"[Text Word] OR "norfolk island"[Text Word] OR "somalia"[Text Word] OR "south africa"[Text Word] OR "south sudan"[Text Word] OR "sri lanka"[Text Word] OR "ceylon"[Text Word] OR "saint kitts and nevis"[Text Word] OR "st kitts and nevis"[Text Word] OR "saint lucia"[Text Word] OR "st lucia"[Text Word] OR "saint vincent"[Text Word] OR "st vincent"[Text Word] OR "grenadines"[Text Word] OR "sudan"[Text Word] OR "suriname"[Text Word] OR "surinam"[Text Word] OR "syria"[Text Word] OR "syrian arab republic"[Text Word] OR "tajikistan"[Text Word] OR "tadjikistan"[Text Word] OR "tadzhikistan"[Text Word] OR "tadzhik"[Text Word] OR "tanzania"[Text Word] OR "tanganyika"[Text Word] OR "thailand"[Text Word] OR "siam"[Text Word] OR "timor leste"[Text Word] OR "east timor"[Text Word] OR "togo"[Text Word] OR "togolese republic"[Text Word] OR "tonga"[Text Word] OR "trinidad"[Text Word] OR "tobago"[Text Word] OR "tunisia"[Text Word] OR "turkey"[Text Word] OR "turkmenistan"[Text Word] OR "turkmen"[Text Word] OR "uganda"[Text Word] OR "ukraine"[Text Word] OR "uruguay"[Text Word] OR "uzbekistan"[Text Word] OR "uzbek"[Text Word] OR "vanuatu"[Text Word] OR "new hebrides"[Text Word] OR "venezuela"[Text Word] OR "vietnam"[Text Word] OR "viet nam"[Text Word] OR "middle east"[Text Word] OR "west bank"[Text Word] OR "gaza"[Text Word] OR "palestine"[Text Word] OR "yemen"[Text Word] OR "yugoslavia"[Text Word] OR "zambia"[Text Word] OR "zimbabwe"[Text Word] OR "northern rhodesia"[Text Word] OR "global south"[Text Word] OR "africa south of the sahara"[Text Word] OR "sub saharan africa"[Text Word] OR "subsaharan africa"[Text Word] OR "central africa"[Text Word] OR "north africa"[Text Word] OR "northern africa"[Text Word] OR "magreb"[Text Word] OR "maghrib"[Text Word] OR "sahara"[Text Word] OR "southern africa"[Text Word] OR "east africa"[Text Word] OR "eastern africa"[Text Word] OR "west africa"[Text Word] OR "western africa"[Text Word] OR "west indies"[Text Word] OR "indian ocean islands"[Text Word] OR "caribbean"[Text Word] OR "central america"[Text

Word] OR "latin america"[Text Word] OR "south america"[Text Word] OR "central asia"[Text Word] OR "north asia"[Text Word] OR "northern asia"[Text Word] OR "southeastern asia"[Text Word] OR "south eastern asia"[Text Word] OR "southeast asia"[Text Word] OR "south east asia"[Text Word] OR "western asia"[Text Word] OR "east europe"[Text Word] OR "eastern europe"[Text Word] OR "developing country"[Text Word] OR "developing countries"[Text Word] OR "developing nation"[Text Word] OR "developing nations"[Text Word] OR "developing population"[Text Word] OR "developing populations"[Text Word] OR "developing world"[Text Word] OR "less developed country"[Text Word] OR "less developed countries"[Text Word] OR "less developed nation"[Text Word] OR "less developed nations"[Text Word] OR "less developed world"[Text Word] OR "lesser developed countries"[Text Word] OR "lesser developed nations"[Text Word] OR "under developed country"[Text Word] OR "under developed countries"[Text Word] OR "under developed nations"[Text Word] OR "under developed world"[Text Word] OR "underdeveloped country"[Text Word] OR "underdeveloped countries"[Text Word] OR "underdeveloped nation"[Text Word] OR "underdeveloped nations"[Text Word] OR "underdeveloped population"[Text Word] OR "underdeveloped populations"[Text Word] OR "underdeveloped world"[Text Word] OR "middle income country"[Text Word] OR "middle income countries"[Text Word] OR "middle income nation"[Text Word] OR "middle income nations"[Text Word] OR "middle income population"[Text Word] OR "middle income populations"[Text Word] OR "low income country"[Text Word] OR "low income countries"[Text Word] OR "low income nation"[Text Word] OR "low income nations"[Text Word] OR "low income population"[Text Word] OR "low income populations"[Text Word] OR "lower income country"[Text Word] OR "lower income countries"[Text Word] OR "lower income nations"[Text Word] OR "lower income population"[Text Word] OR "lower income populations"[Text Word] OR "underserved countries"[Text Word] OR "underserved nations"[Text Word] OR "underserved population"[Text Word] OR "underserved populations"[Text Word] OR "under served population"[Text Word] OR "under served populations"[Text Word] OR "deprived countries"[Text Word] OR "deprived population"[Text Word] OR "deprived populations"[Text Word] OR "poor country"[Text Word] OR "poor countries"[Text Word] OR "poor nation"[Text Word] OR "poor nations"[Text Word] OR "poor population"[Text Word] OR "poor populations"[Text Word] OR "poor world"[Text Word] OR "poorer countries"[Text Word] OR "poorer nations"[Text Word] OR "poorer population"[Text Word] OR "poorer populations"[Text Word] OR "developing economy"[Text Word] OR "developing economies"[Text Word] OR "less developed economy"[Text Word] OR "less developed economies"[Text Word] OR "underdeveloped economies"[Text Word] OR "middle income economy"[Text Word] OR "middle income economies"[Text Word] OR "low income economy"[Text Word] OR "low income economies"[Text Word] OR "lower income economies"[Text Word] OR "low gdp"[Text Word] OR "low gnp"[Text Word] OR "low gross domestic"[Text Word] OR "low gross national"[Text Word] OR "lower gdp"[Text Word] OR "lower gross domestic"[Text Word] OR "lmic"[Text Word] OR "lmics"[Text Word] OR "third world"[Text Word] OR "lami

country"[Text Word] OR "lami countries"[Text Word] OR "transitional country"[Text Word] OR "transitional countries"[Text Word] OR "emerging economies"[Text Word] OR "emerging nation"[Text Word] OR "emerging nations"[Text Word])))

CINAHL

(MH "Multidisciplinary Care Team+") OR (MM "Rural Health Personnel") OR (MH "Health Personnel+") OR (MH "Community Health Workers") OR ("chw*") OR (MH "Association of Community Health Nursing Educators") OR (MH "Community Practitioners' and Health Visitors' Association") OR (MM "Community Health Workers") OR (Non-physician*) OR (health auxiliary) OR (physician assistant*) OR (MH "Physician Assistants+") OR (“Mid-level health provider*”) OR (MH “nurse*”) OR (licensed practical nurse) OR (nurse midwife) OR (nurse*) OR (registered nurse*) OR (“health extension worker*”) OR (MH "Dental Auxiliaries+") OR (MH "Practical Nurses") OR (“auxiliary nurse*”) OR (MH "Medical Assistants") OR (MH "Students, Physician Assistant") OR (MH "Physician Assistants") OR (“medical assistant*”) OR (“clinical associate*”) OR (“task shift*”) OR (“task shar*”)

AND

(“chronic disease*”) OR (“non communicable disease*”) OR (diabetes mellitus) OR (“ncd*”) OR (“cardiovascular risk*”) OR (“cardiovascular disease*”) OR (“cvd*”) OR (MH "Hypertension+") OR (MH "Hypertension, White Coat") OR (MH "Hypertension, Renovascular") OR (MH "Hypertension, Refractory") OR (MH "Pulmonary Arterial Hypertension") OR (MH "Hypertension, Pulmonary+") OR (MH "Hypertension, Isolated Systolic") OR (MH "Pregnancy-Induced Hypertension+") OR (MH "Ocular Hypertension+") OR (MH "Masked Hypertension") OR (MH "Intracranial Hypertension+") OR (MH "Hypertension, Renal+") OR (MH "Hypertension, Portal+") OR (“resistant hypertension”) OR (“systolic hypertension”) OR (“hereditary hypertension”) OR (“white coat hypertension”) OR (“borderline hypertension”) OR (“maternal hypertension”) OR (“diabetic hypertension”) OR (“malignant hypertension”) OR (“essential hypertension”) OR (“renovascular hypertension”) OR (MH "Diabetes Mellitus, Type 1+") OR (MH "Diabetes Mellitus, Type 2") OR (MH "Diabetes Mellitus, Gestational") OR (MH "Pregnancy in Diabetes+") OR (MH "Diabetes Mellitus+") OR (MH "Diabetes Educators") OR (MH "Diabetes Education") OR (MH "Diabetic Patients") OR (MH "Glycemic Control") OR (“diabetes mellitus”) OR (MH "Cancer Fatigue") OR (MH "Prostatic Neoplasms, Castration-Resistant") OR (MH "Early

Detection of Cancer") OR (MH "Cancer Survivors") OR (MH "Cancer Screening") OR (MH "Cancer Vaccines+") OR (MH "Cancer Patients") OR (MH "Vulvar Neoplasms") OR (MH "Neoplasms, Germ Cell and Embryonal+") OR (MH "Cancer-Associated Fibroblasts") OR (MH "Neoplasm Metastasis+") OR (“cancer*”) OR (“neoplasm*”) OR (“malignan*”) OR (MH "Neoplasms, Ductal, Lobular, and Medullary+") OR (MH "Hematologic Neoplasms+") OR (MH "Neoplasms, Cystic, Mucinous, and Serous+") OR (MH "Neoplasms, Germ Cell and Embryonal+") OR (MH "Neoplasms, Glandular and Epithelial+") OR (MH "Neoplasm Regression, Spontaneous") OR (MH "Meningeal Neoplasms+") OR (MH "Neoplasms, Adnexal and Skin Appendage") OR (MH "Neoplasm Staging") OR (MH "Neuroectodermal Tumors, Primitive+") OR (MH "Neoplasms, Connective and Soft Tissue+") OR (MH "Neoplasms, Nerve Tissue+") OR (MH "Infratentorial Neoplasms+") OR (MH "Hypothalamic Neoplasms+") OR (MH "Neoplasm Grading") OR (MH "Respiratory Tract Diseases+") OR (MH "Respiratory Hypersensitivity+") OR (MH "Respiratory Tract Neoplasms+") OR (“respiratory disease”) OR (“extrinsic asthma”) OR (“exercise induced asthma”) OR (“severe persistent asthma”) OR (“asthma”) OR (“mild intermittent asthma”) OR (“occupational asthma”) OR (“eosinophilic asthma”) OR (“allergic asthma”) OR (“nocturnal asthma”) OR (“intrinsic asthma”) OR (“moderate persistent asthma”) OR (“mild persistent asthma”) OR (MH "Asthma+") OR (MM "Asthma, Occupational") OR (MM "Asthma-Chronic Obstructive Pulmonary Disease Overlap Syndrome") OR (MM "Asthma, Exercise-Induced") OR (“cardiac asthma”) OR (“severe asthma”) OR (MH "Pulmonary Disease, Chronic Obstructive+") OR (MH "Lung Diseases, Obstructive+") OR (MH "Chronic Disease+") OR (“COPD*”) OR (“mental health”) OR (“anxiety assessment”) OR (“anxiety neurosis”) OR (“anxiety disorder”) OR (“anxiety”) OR (“generalized anxiety disorder”) OR (MH "anxiety+") OR (“post-stroke depression”) OR ("mixed mania and depression") OR (recurrent brief depression) OR (inbreeding depression) OR (“treatment resistant depression”) OR (“postoperative depression”) OR (“depression”) OR (“bipolar depression”) OR (“masked depression”) OR (“minor depression”) OR (“involutional depression”) OR (“depression assessment”) OR (“atypical depression”) OR (“adolescent depression”) OR (“long term depression”) OR (“postnatal depression”) OR (“endogenous depression”) OR ("mixed depression and dementia") OR (“late life depression”) or (“antenatal depression”) OR (“organic depression”) OR (“perinatal depression”) OR (“reactive depression”) OR (“chronic

depression”) OR (“agitated depression”) OR (“major depression”) OR ("mixed anxiety and depression") OR (MH "depression+")

((afghanistan OR albania OR algeria OR "american samoa" OR angola OR "antigua and barbuda" OR antigua OR barbuda OR argentina OR armenia OR armenian OR aruba OR azerbaijan OR bahrain OR bangladesh OR barbados OR "republic of belarus" OR belarus OR byelarus OR belorussia OR byelorussian OR belize OR "british honduras" OR benin OR dahomey OR bhutan OR bolivia OR "bosnia and herzegovina" OR bosnia OR herzegovina OR botswana OR bechuanaland OR brazil OR brasil OR bulgaria OR "burkina faso" OR "burkina fasso" OR "upper volta" OR burundi OR urundi OR "cabo verde" OR "cape verde" OR cambodia OR kampuchea OR "khmer republic" OR cameroon OR cameron OR cameroun OR "central african republic" OR "ubangi shari" OR chad OR chile OR china OR colombia OR comoros OR "comoro islands" OR "iles comores" OR mayotte OR "democratic republic of the congo" OR "democratic republic congo" OR congo OR zaire OR "costa rica" OR "cote d’ivoire" OR "cote d’ ivoire" OR "cote divoire" OR "cote d ivoire" OR "ivory coast" OR croatia OR cuba OR cyprus OR "czech republic" OR czechoslovakia OR djibouti OR "french somaliland" OR dominica OR "dominican republic" OR ecuador OR egypt OR "united arab republic" OR "el salvador" OR "equatorial guinea" OR "spanish guinea" OR eritrea OR estonia OR eswatini OR swaziland OR ethiopia OR fiji OR gabon OR "gabonese republic" OR gambia OR "georgia (republic)" OR georgia OR georgian OR ghana OR "gold coast" OR gibraltar OR greece OR grenada OR guam OR guatemala OR guinea OR "guinea bissau" OR guyana OR "british guiana" OR haiti OR hispaniola OR honduras OR hungary OR india OR indonesia OR timor OR iran OR iraq OR "isle of man" OR jamaica OR jordan OR kazakhstan OR kazakh OR kenya OR "democratic people’s republic of korea" OR "republic of korea" OR north korea OR south korea OR korea OR kosovo OR kyrgyzstan OR kirghizia OR kirgizstan OR "kyrgyz republic" OR kirghiz OR laos OR "lao pdr" OR "lao people's democratic republic" OR latvia OR lebanon OR "lebanese republic" OR lesotho OR basutoland OR liberia OR libya OR "libyan arab jamahiriya" OR lithuania OR macau OR macao OR "republic of north macedonia" OR macedonia OR madagascar OR "malagasy republic" OR malawi OR nyasaland OR malaysia OR "malay federation" OR "malaya federation" OR maldives OR "indian ocean islands" OR "indian ocean" OR mali OR malta OR micronesia OR "federated states of micronesia" OR kiribati OR "marshall islands" OR nauru OR "northern mariana islands" OR palau OR tuvalu OR mauritania OR mauritius OR

mexico OR moldova OR moldovian OR mongolia OR montenegro OR morocco OR ifni OR mozambique OR "portuguese east africa" OR myanmar OR burma OR namibia OR nepal OR "netherlands antilles" OR nicaragua OR niger OR nigeria OR oman OR muscat OR pakistan OR panama OR "papua new guinea" OR paraguay OR peru OR philippines OR philipines OR phillipines OR phillippines OR poland OR "polish people's republic" OR portugal OR "portuguese republic" OR "puerto rico" OR romania OR russia OR "russian federation" OR ussr OR "soviet union" OR "union of soviet socialist republics" OR rwanda OR ruanda OR samoa OR "pacific islands" OR polynesia OR "samoan islands" OR "navigator island" OR "navigator islands" OR "sao tome and principe" OR "saudi arabia" OR senegal OR serbia OR seychelles OR "sierra leone" OR slovakia OR "slovak republic" OR slovenia OR melanesia OR "solomon island" OR "solomon islands" OR "norfolk island" OR "norfolk islands" OR somalia OR "south africa" OR "south sudan" OR "sri lanka" OR ceylon OR "saint kitts and nevis" OR "st. kitts and nevis" OR "saint lucia" OR "st. lucia" OR "saint vincent and the grenadines" OR "saint vincent" OR "st. vincent" OR grenadines OR sudan OR suriname OR surinam OR "dutch guiana" OR "netherlands guiana" OR syria OR "syrian arab republic" OR tajikistan OR tadjikistan OR tadzhikistan OR tadzhik OR tanzania OR tanganyika OR thailand OR siam OR "timor leste" OR "east timor" OR togo OR "togolese republic" OR tonga OR "trinidad and tobago" OR trinidad OR tobago OR tunisia OR turkey OR turkmenistan OR turkmen OR uganda OR ukraine OR uruguay OR uzbekistan OR uzbek OR vanuatu OR "new hebrides" OR venezuela OR vietnam OR "viet nam" OR "middle east" OR "west bank" OR gaza OR palestine OR yemen OR yugoslavia OR zambia OR zimbabwe OR "northern rhodesia" OR "global south" OR "africa south of the sahara" OR "sub saharan africa" OR "subsaharan africa" OR "africa, central" OR "central africa" OR "africa, northern " " OR "north africa" OR "northern africa" OR magreb OR maghrib OR sahara OR "africa, southern " " OR "southern africa" OR "africa, eastern " " OR "east africa" OR "eastern africa" OR "africa, western " " OR " " west africa " " OR " " western africa " " OR "west indies" OR "indian ocean islands" OR caribbean OR "central america" OR "latin america" OR "south and central america" OR "south america" OR "asia, central" OR "central asia" OR "asia, northern" OR "north asia" OR "northern asia" OR "asia, southeastern" OR "southeastern asia" OR "south eastern asia" OR "southeast asia" OR "south east asia" OR "asia, western" OR "western asia" OR "europe, eastern" OR "east europe" OR "eastern europe" OR "developing country" OR "developing

countries" OR "developing nation" OR "developing nations" OR "developing population" OR "developing populations" OR "developing world" OR "less developed country" OR "less developed countries" OR "less developed nation" OR "less developed nations" OR "less developed population" OR "less developed populations" OR "less developed world" OR "lesser developed country" OR "lesser developed countries" OR "lesser developed nation" OR "lesser developed nations" OR "lesser developed population" OR "lesser developed populations" OR "lesser developed world" OR "under developed country" OR "under developed countries" OR "under developed nation" OR "under developed nations" OR "under developed population" OR "under developed populations" OR "under developed world" OR "underdeveloped country" OR "underdeveloped countries" OR "underdeveloped nation" OR "underdeveloped nations" OR "underdeveloped population" OR "underdeveloped populations" OR "underdeveloped world" OR "middle income country" OR "middle income countries" OR "middle income nation" OR "middle income nations" OR "middle income population" OR "middle income populations" OR "low income country" OR "low income countries" OR "low income nation" OR "low income nations" OR "low income population" OR "low income populations" OR "lower income country" OR "lower income countries" OR "lower income nation" OR "lower income nations" OR "lower income population" OR "lower income populations" OR "underserved country" OR "underserved countries" OR "underserved nation" OR "underserved nations" OR "underserved population" OR "underserved populations" OR "underserved world" OR "under served country" OR "under served countries" OR "under served nation" OR "under served nations" OR "under served population" OR "under served populations" OR "under served world" OR "deprived country" OR "deprived countries" OR "deprived nation" OR "deprived nations" OR "deprived population" OR "deprived populations" OR "deprived world" OR "poor country" OR "poor countries" OR "poor nation" OR "poor nations" OR "poor population" OR "poor populations" OR "poor world" OR "poorer country" OR "poorer countries" OR "poorer nation" OR "poorer nations" OR "poorer population" OR "poorer populations" OR "poorer world" OR "developing economy" OR "developing economies" OR "less developed economy" OR "less developed economies" OR "lesser developed economy" OR "lesser developed economies" OR "under developed economy" OR "under developed economies" OR "underdeveloped economy" OR "underdeveloped economies" OR "middle income economy" OR "middle income economies" OR "low income economy" OR "low income

economies" OR "lower income economy" OR "lower income economies" OR "low gdp" OR "low gnp" OR "low gross domestic" OR "low gross national" OR "lower gdp" OR "lower gnp" OR "lower gross domestic" OR "lower gross national" OR lmic OR lmics OR "third world" OR "lami country" OR "lami countries" OR "transitional country" OR "transitional countries" OR "emerging economies" OR "emerging nation" OR "emerging nations")) OR ((afghan OR afghans OR afghani OR albanian OR albanians OR algerian OR algerians OR "american samoan" OR "american samoans" OR angolan OR angolans OR antiguan OR antiguans OR barbudan OR berbudans OR argentine OR argentines OR argentinian OR argentinians OR argentinean OR argentineans OR armenian OR armenians OR aruban OR arubans OR azerbaijani OR azerbaijanis OR bahraini OR bahrainis OR bangladeshi OR bangladeshis OR bangalees OR bajan OR bajans OR belarusian OR belarusians OR byelorussian OR byelorussians OR belizean OR belizeans OR beninese OR benineses OR bhutanese OR bolivian OR bolivians OR bosnian OR bosnians OR botswana OR batswana OR brazilian OR brazilians OR brasilian OR brasilians OR bulgarian OR bulgarians OR burkinabe OR burkinese OR burundian OR burundians OR "cape verdean" OR "cape verdeans" OR "cabo verdean" OR "cabo verdeans" OR cambodian OR cambodians OR khmer OR cameroonian OR cameroonians OR "central african" OR "central africans" OR chadian OR chadians OR chilean OR chileans OR chinese OR colombian OR colombians OR comorian OR comorians OR congolese OR "costa rican" OR "costa ricans" OR ivorian OR ivorians OR croatian OR croatians OR cuban OR cubans OR cypriot OR cypriots OR czech OR czechs OR djiboutian OR djiboutians OR dominican OR dominicans OR ecuadorian OR ecuadorians OR egyptian OR egyptians OR salvadoran OR salvadorans OR "equatorial guinean" OR "equatorial guineans" OR equatoguinean OR equatoguineans OR eritrean OR eritreans OR estonian OR estonians OR swazi OR swazis OR swati OR swatis OR ethiopian OR ethiopians OR fijian OR fijians OR gabonese OR gabonaise OR gambian OR gambians OR georgian OR georgians OR ghanaian OR ghanaians OR gibraltarian OR gibraltarians OR greek OR greeks OR grenadian OR grenadians OR guamanian OR guamanians OR guatemalan OR guatemalans OR guinean OR guineans OR "bissau guinean" OR "bissau guineans" OR guyanese OR haitian OR haitians OR honduran OR hondurans OR hungarian OR hungarians OR indian OR indians OR indonesian OR indonesians OR iranian OR iranians OR iraqian OR iraqians OR iraqi OR iraqis OR manx OR jamaican OR jamaicans OR jordanian OR jordanians OR

kazakhstani OR kazakhstanis OR kenyan OR kenyans OR kirabati OR kirabatian OR kirabatians OR "north korean" OR "north koreans" OR korean OR koreans OR kosovar OR kosovars OR kosovan OR kosovans OR kyrgyzstani OR kyrgyzstanis OR kyrgyz OR lao OR laotian OR laotians OR latvian OR latvians OR lebanese OR lesothan OR lesothans OR lesothonian OR lesothonians OR mosotho OR basotho OR liberian OR liberians OR libyan OR libyans OR lithuanian OR lithuanians OR macanese OR macedonian OR macedonians OR malagasy OR madagascan OR madagascans OR malawian OR malawians OR malaysian OR malaysians OR maldivian OR maldivians OR malian OR malians OR maltese OR marshallese OR marshalleses OR mauritanian OR mauritanians OR mauritian OR mauritians OR mexican OR mexicans OR micronesian OR micronesians OR moldovan OR moldovans OR mongolian OR mongolians OR mongol OR montenegrin OR montenegrins OR moroccan OR moroccans OR mozambican OR mozambicans OR burmese OR myanma OR namibian OR namibians OR nauruan OR nauruans OR nepali OR nepalese OR "netherlands antillean" OR "netherlands antilleans" OR nicaraguan OR nicaraguans OR nigerien OR nigeriens OR nigerian OR nigerians OR "northern mariana islander" OR "northern mariana islanders" OR mariana OR marianas OR omani OR omanis OR pakistani OR pakistanis OR palauan OR palauans OR panamanian OR panamanians OR "papua new guinean" OR "papua new guineans" OR paraguayan OR paraguayans OR peruvian OR peruvians OR philippine OR philippines OR philipine OR philipines OR phillipine OR phillipines OR phillippine OR phillippines OR filipino OR filipinos OR filipina OR filipinas OR polish OR pole OR poles OR portuguese OR "puerto rican" OR "puerto ricans" OR romanian OR romanians OR russian OR russians OR "soviet people" OR "soviet population" OR rwandan OR rwandans OR rwandese OR ruandan OR ruandans OR ruandese OR samoan OR samoans OR "sao tomean" OR "sao tomeans" OR santomean OR santomeans OR "saudi arabian" OR "saudi arabians" OR saudi OR saudis OR senegalese OR serbian OR serbians OR montenegrin OR montenegrins OR seychellois OR seychelloise OR seychelloises OR "sierra leonean" OR "sierra leoneans" OR slovak OR slovaks OR slovene OR slovenes OR "solomon islander" OR "solomon islanders" OR somali OR somalis OR "south african" OR "south africans" OR "south sudanese" OR "sri lankan" OR "sri lankans" OR ceylonese OR kittitian OR kittitians OR nevisian OR nevisians OR "saint lucian" OR "saint lucians" OR vincentian OR vincentians OR sudanese OR surinamese OR surinameses OR syrian OR syrians OR tajik OR tajiks OR tajikistani OR tajikistanis

OR tanzanian OR tanzanians OR tanganyikan OR tanganyikans OR thai OR timorese OR timoreses OR togolese OR tongan OR tongans OR trinidadian OR trinidadians OR tobagonian OR tobagonians OR tunisian OR tunisians OR turk OR turks OR turkish OR turkmen OR turkmens OR tuvaluan OR tuvaluans OR ugandan OR ugandans OR ukrainian OR ukrainians uruguayan OR uruguayans OR uzbek OR uzbeks OR vanuatu OR vanuatuan OR vanuatuans OR venezuelan OR venezuelans OR vietnamese OR yemeni OR yemenis OR yemenite OR yemenites OR yemenese OR yugoslav OR yugoslavs OR yugoslavian OR yugoslavians OR zambian OR zambians OR zimbabwean OR zimbabweans))
