# Supplementary material for: Task-sharing for non-communicable disease prevention and control in low- and middle-income countries in the context of health worker shortages: A systematic review
Source: PLOS Glob Public Health. 2025 Apr 16;5(4):e0004289. doi: 10.1371/journal.pgph.0004289 (PMC12002516; doi:10.1371/journal.pgph.0004289)
Supplement: S2 Appendix – — Risk of bias results. (PDF) [file pgph.0004289.s002.pdf]

S2 Appendix – Risk of bias results

	Randomisation	Deviations from intended interventions	Missing outcome data	Measurement of the outcome	Selection of reported result	Overall risk of bias
**Low**	115	120	129	133	131	85
**Some**	29	27	19	13	16	52
**High**	5	2	1	3	2	12
