# Supplementary material for: Task-sharing for non-communicable disease prevention and control in low- and middle-income countries in the context of health worker shortages: A systematic review
Source: PLOS Glob Public Health. 2025 Apr 16;5(4):e0004289. doi: 10.1371/journal.pgph.0004289 (PMC12002516; doi:10.1371/journal.pgph.0004289)
Supplement: S3 Appendix – — Evidence of effective task-sharing from this review. (PDF) [file pgph.0004289.s003.pdf]

S3 Appendix – Evidence of effective task-sharing from this review

A. Cervical Cancer

Cancer care services		Community	Lay counsellor	Traditional; faith healer	CHW	Care coordinator	Nurse	Midwife	Doctor	Pharmacy technician or Pharmacist	Dietician
Prevention	Education & Promotion				Yes(33) (17, 44)						
	Vaccine administration										
	VIA screening				Yes(17)		Yes(127)				
Diagnosis	Test kit delivery				Yes(33)						
	Sample self-collection	Yes,(33, 44)									
	Sample delivery to HPV lab				Yes(33)						

B. GIT Cancers

Cancer care services		Community	Lay counsellor	Traditional and faith healer	CHW	Care coordinator	Nurse	Midwife	Doctor	Pharmacy technician and Pharmacist	Dietician
Prevention	Education (i.e. phone-based MI counseling)						Yes(124)				
	Screening						Yes(190)				
Diagnosis	Sample self-collection	Yes(141)									

C. Breast cancer

Cancer care services		Community	Lay counsellors	Traditional healer	CHW	Care coordinator	Nurse	Midwife	Doctor	Pharmacist	Dietician
Prevention	Education				Yes(16, 63, 93, 109)		Yes(21, 62, 130)				
	Screening				Yes (16, 63, 109)		Yes (109, 115)	Yes(115)			
Diagnosis (Needle biopsy)									Yes (109, 115)		
Treatment	Chemotherapy										
	Radiotherapy										
	Adherence										
Palliation											
Rehabilitation		Yes(62)					Yes(42)				

D. Mental Health

Mental Health		Community	CHWs	Traditional and faith healers	Psychologist /Social workers	Care coordinator	Physiotherapist	Nurse	Health officers	Doctor	Specialist doctor
Prevention	Education	Yes (87)	Yes(37, 95, 136, 142, 148, 149, 191)		Yes (95)			Yes (87, 116, 192)			
	Screening	Yes (87)	Yes(50, 66, 95, 108, 111, 112, 117, 149)		Yes (95, 191)			Yes (86, 87, 175, 193, 194)			
Diagnosis			Yes (108)					Yes (29, 87, 110, 142)		Yes (46, 50) ✓(29)	Yes (193) (29)
Treatment	Monitoring comorbidities		Yes (118, 149)			Yes (31)		Yes (34, 116)		Yes (46)	
	Support for self-care		Yes (23, 24, 35, 38, 39, 51, 66, 95, 111, 112, 114, 118, 142, 149, 175, 195)	Yes (25)	Yes (50, 84, 95, 134)	Yes (31)		Yes (34, 40, 84, 86, 98, 116, 123, 126, 134, 142, 175, 192, 193, 196)		Yes (142, 197) (193)	Yes (31, 195)
	Problem-solving therapy and		Yes (23, 24, 35, 38, 50, 51, 66, 108, 111, 112, 118, 142, 148,	Yes (25)	Yes (50, 84, 95)			Yes (40, 51, 110, 123, 196, 197) (29) (25,	Yes (23)		Yes (134)

	psychoeducation		191) (24, 95, 195)					40, 86, 126, 134)			
	Adherence		Yes (35, 39, 66, 117, 118, 149, 151, 175, 195)	Yes (25)				Yes (34, 98, 116, 123, 142, 193, 197)			Yes (195)
	Behaviour change		Yes (29, 37, 114, 142)		Yes (84)			Yes (34, 116, 192, 197)			
	Prescribing							Yes (25, 175, 194)	Yes (23)		
	Injections							Yes (192)			
	Prevention of complications		Yes (39, 114, 117) (117, 148)					Yes (34, 123) (194)			
Referral			Yes (35, 38, 50, 51, 114, 117, 118, 136, 148, 149, 195)			Yes (31)		Yes (84, 98, 110-112, 116, 123, 175, 194) (34, 116)	Yes (23)		
Rehabilitation					Yes (134)		Yes (134)	Yes (134)			

E. Diabetes

Diabetes		Community	CHWs	Psychologist	Care coordinator	Dietician	Junior Nurse	Nurse	Doctor	Specialist doctor
Prevention	Education	Yes (67)	Yes (55, 67, 76, 149)		Yes (31)					
	Screening		Yes (67, 76, 149)					Yes (76)		
Diagnosis	Making diagnosis							Yes (92)	✓ (92)	✓ (92) (31)
	understand diagnosis	Yes (67)	Yes (53, 55, 67, 74, 149, 176)	Yes (75)		Yes (94, 103, 139)	Yes (120)	Yes (15, 34, 52, 92, 129, 161)		
Treatment	Monitoring of blood glucose		Yes (53, 67, 74, 76)		Yes (31)		Yes (120)	Yes (15, 34, 52, 102, 129, 138, 161, 176)		
	Support for self-care or counselling		Yes (53, 55, 67, 74, 176)	Yes (75)	Yes (31)	Yes (139)	Yes (120)	Yes (15, 34, 52, 92, 102, 129, 138, 161)		Yes (31)
	Create individualised care plan		Yes (149, 176)			Yes (94, 139)		Yes (15, 52, 75, 102)		
	Adherence		Yes(53, 55, 67, 74, 176)	Yes(75)	Yes (31)		Yes (120)	Yes(34, 129, 161)		Yes (31)
	Behaviour change	Yes (67)	Yes (67, 74, 76)	Yes (75)	Yes (31)	Yes (94, 103, 139)		Yes (15, 34, 52, 75, 102, 129, 138, 161)		
	Prescription of medication							Yes (52, 57)	Yes (57, 75, 92, 138)	Yes (31, 57, 92)
	Prevention of complications	Yes (67)	Yes (53, 74, 176)		Yes (31, 53)			Yes (34, 52, 57, 92, 102, 129)		Yes (31, 52, 92)
Referral			Yes (53, 149, 176)		Yes (31)			Yes (52, 57)	Yes (75)	

F. Heart Failure

Heart Failure		Community	Lay counsellors	Traditional healer	CHWs	Care coordinator	Nurse	Midwife	Doctor	Pharmacist	Dietician/ Nutritionist
Prevention	Education										
	Screening										
Diagnosis											
Treatment	Adherence						Yes(155)				
	Behaviour change	Yes(135)					Yes(135)				
	Prevention of complications						Yes(54)				
Rehabilitation	Cardiac rehabilitation						Yes(32, 54, 100, 162)				

G. Ischaemic Heart disease

Heart disease services		Community	Lay counsellors	Traditional healers	CHWs	Care coordinator	Nurse	Midwife	Doctor	Pharmacist	Dietician/ Nutritionists
**Prevention**	Education						Yes(60, 70, 152)				
	Screening				Yes(36)						
**Diagnosis**											
**Treatment**	Adherence				Yes(132)		Yes(78)				
	Behaviour change						Yes(78, 79)				
	Prevention of complications						Yes(153, 158, 198)				
**Rehabilitation**											

H. Hypertension

Heart disease services		Community	Lay counsellors	Traditional healers	CHWs	Care coordinator	Nurse	Doctor	Pharmacist	Dietician/Nutritionists
Prevention	Education				Yes(43, 72, 105)		Yes			
	Screening				Yes (27, 113, 144, 164)					
Diagnosis										
Treatment	Treatment initiation						Yes(168, 180)			
	Adherence				Yes(27, 64, 65, 105, 113, 144, 164, 174, 180)		Yes(30, 64, 71-73, 199, 200)			
	Behaviour change				Yes (58, 65, 73, 105, 113, 144, 174)		Yes(58, 71-73, 80, 107, 113, 116, 131, 154, 160, 180, 200, 201)			
	Referral				Yes(113, 168, 180)		Yes(28, 58, 80, 113, 116)			
	Prevention of complications						Yes			

I. Cardiovascular risk

CVD Risk		Community Network Teams	CHWs	Lay counsellors	Traditional and faith healers	Care coordinator	Pharmacy technicians	Dietician/ Nutritionist	Nurse	Midwife	Doct or
Preventio n	Primary prevention (education)		Yes(76, 80, 88)						Yes (68, 158)		
	Screening		Yes(27, 28, 80, 113, 202)								
Diagnosis											
Treatment	Adherence		Yes(27, 80)								
	Lifestyle changes		Yes (27, 28, 80)								
	Prevention of complications										
