# Supplementary material for: Task-sharing for non-communicable disease prevention and control in low- and middle-income countries in the context of health worker shortages: A systematic review
Source: PLOS Glob Public Health. 2025 Apr 16;5(4):e0004289. doi: 10.1371/journal.pgph.0004289 (PMC12002516; doi:10.1371/journal.pgph.0004289)
Supplement: Attachment — Submitted filename: Response to Editor_Task-sharing for NCDs.pdf [file pgph.0004289.s005.pdf]

University of New South Wales (UNSW)
Tel : +61 435038705
E-mail : a.tesema@unsw.edu.au

21 December 2024

Author Response Letter: PLOS Global Public Health

Dear Dr Roopa Shivashankar

I am writing on behalf of all authors to submit the revised manuscript on "*Task-sharing for non-communicable disease prevention and control in low- and middle-income countries in the context of health worker shortages: A systematic review*". We appreciate the opportunity to respond to the to address the editorial suggestions and have prepared a point-by-point response to your comments. Your suggestions are highlighted in italics, followed by our responses in plain text, presented with bullet points for clarity. We have submitted the required data along with this revision as per the editorial requirements.

Journal Requirements:

As required by our policy on Data Availability, please ensure your manuscript or supplementary information includes the following:

1. *A numbered table of all studies identified in the literature search, with the full title of each article, including those that were excluded from the analyses.*

- Thank you for your feedback.
  We used COVIDENCE software for the systematic screening and management of studies during this review. We were able to download all studies identified in the literature search after the initial screening of titles and abstracts for 4150 articles (3486 citations in Phase 1 and 664 in Phase 2) from Covidence. Additionally, we have provided a table listing the studies excluded from the analysis as well as those that were finally included in the study. Please refer to the files titled *'All studies identified in the literature search_Phase 1'* and *'All studies identified in the literature search_Phase 2.'*

2. *A table of all data extracted from the primary research sources for the systematic review and/or meta-analysis.*
    - Thank you. We have uploaded a table containing all data extracted from the primary research sources for the study. Please refer to the file titled *'Final data extracted from the primary research sources_phases 1&2'.*

3. *A table showing the completed risk of bias and quality/certainty assessments for each study or outcome.*
    - Thank you. We have uploaded a completed table assessing the risk of bias for the study. Please refer to the file titled *'Final completed risk of bias_phases 1 &2'.*

Thank you again for your feedback and for considering our revised paper. Please do not hesitate to contact me if you have any further questions.

Best regards,

Azeb Gebresilassie Tesema
