# Supplementary material for: Task-sharing for non-communicable disease prevention and control in low- and middle-income countries in the context of health worker shortages: A systematic review
Source: PLOS Glob Public Health. 2025 Apr 16;5(4):e0004289. doi: 10.1371/journal.pgph.0004289 (PMC12002516; doi:10.1371/journal.pgph.0004289)
Supplement: Attachment — Submitted filename: Feedback_PGPH-D-24-02123.docx [file pgph.0004289.s004.docx]

1. **Background:**

- When there is shortage of already health workers then how tasks can be shifted to them? What is rationales for shifting tasks to NPHWs? If author could justify with evidence as the ratio of availability of physicians and NPHWs in LMICs then this task shifting becomes more relevant
- Additionally, it would be helpful to include a brief review of why task-sharing is particularly important for NCD management in LMICs e.g. decentralization of services, patient retention in care

2. **Methods**

- The study included many different types of interventions and health workers (like nurses and community health workers) but didn't analyse any subgroups. To make the study stronger, the researchers should look at subgroups separately. For example, they could see if certain types of non-physician health workers were better at providing specific services, like mental health care versus care for heart disease. This would help determine if certain types of health workers are more effective in certain situations. This information is available in Table 2, but analysing it more closely would strengthen the study's findings.

3. **Results:**

- The manuscript relies too heavily on text and lacks visual aids. It would be easier to understand if it included visuals like pie charts or bar graphs to show the distribution of studies across different regions, diseases, or types of health workers. For example, a bar graph could effectively illustrate the effectiveness of task-sharing across different disease categories, such as mental health and cardiovascular disease.

4. **Discussions:**

- Insufficient Analysis of Barriers: The discussion on barriers (e.g., non-availability of medicines, long waiting times) is brief and lacks depth. The manuscript should delve deeper into how these barriers directly affected the outcomes. Were there studies where interventions failed due to these barriers? Providing case-specific examples would enhance the discussion.
